# Modelling Mixed-Gas Sorption in Glassy Polymers for CO_2_ Removal: A Sensitivity Analysis of the Dual Mode Sorption Model

**DOI:** 10.3390/membranes9010008

**Published:** 2019-01-04

**Authors:** Eleonora Ricci, Maria Grazia De Angelis

**Affiliations:** Department of Civil, Chemical, Environmental and Materials Engineering, University of Bologna, 40131, Bologna, Italy; grazia.deangelis@unibo.it

**Keywords:** dual mode sorption model, mixed-gas sorption, PIMs, glassy polymers

## Abstract

In an effort to reduce the experimental tests required to characterize the mixed-gas solubility and solubility-selectivity of materials for membrane separation processes, there is a need for reliable models which involve a minimum number of adjustable parameters. In this work, the ability of the Dual Mode Sorption (DMS) model to represent the sorption of CO_2_/CH_4_ mixtures in three high free volume glassy polymers, poly(trimethylsilyl propyne) (PTMSP), the first reported polymer of intrinsic microporosity (PIM-1) and tetrazole-modified PIM-1 (TZ-PIM), was tested. The sorption of gas mixtures in these materials suitable for CO_2_ separation has been characterized experimentally in previous works, which showed that these systems exhibit rather marked deviations from the ideal pure-gas behavior, especially due to competitive effects. The accuracy of the DMS model in representing the non-idealities that arise during mixed-gas sorption was assessed in a wide range of temperatures, pressures and compositions, by comparing with the experimental results available. Using the parameters obtained from the best fit of pure-gas sorption isotherms, the agreement between the mixed-gas calculations and the experimental data varied greatly in the different cases inspected, especially in the case of CH_4_ absorbed in mixed-gas conditions. A sensitivity analysis revealed that pure-gas data can be represented with the same accuracy by several different parameter sets, which, however, yield markedly different mixed-gas predictions, that, in some cases, agree with the experimental data only qualitatively. However, the multicomponent calculations with the DMS model yield more reliable results than the use of pure-gas data in the estimation of the solubility-selectivity of the material.

## 1. Introduction

In recent years, the use of polymers as membrane materials has attracted increased interest for several industrial applications, including gas separation for hydrogen recovery, nitrogen production, air dehydration, natural gas sweetening and biogas upgrading [1]. CO_2_ is a typical contaminant to be removed from both natural gas and biomethane, in order to meet distribution pipelines specifications [2]. Despite the fact that CO_2_ removal from natural gas with membranes has found industrial application since the 1980s [3], nowadays this technology has only about 10% of the market, which is dominated by solvent absorption using amines [1]. In membrane materials design research, countless structural and molecular modifications have been investigated in order to achieve a better separation performance, that would make membranes more competitive, in addition to being more energy-efficient and environmentally friendly [4,5,6,7,8,9,10]. However, one of the greatest challenges faced in membrane materials design is the existence of a trade-off between permeability and selectivity: for every gas pair the logarithm of the selectivity versus the logarithm of the permeability of the most permeable gas has been shown to lie below a limiting line, customarily referred to as the Robeson upper bound [11,12]. This is due to the fact that ultra-permeable materials usually display very poor selectivity, whereas highly-selective materials exhibit lower permeabilities [12]. This sets an upper limit to the efficiency that can be achieved by the operation, in case it is governed by diffusivity-selectivity [13,14].

Materials with improved performance, capable of surpassing the upper bound, have nonetheless been developed, among those are the family of Polymers of Intrinsic Microporosity (PIMs) [15,16,17,18] and Thermally Rearranged (TR) polybenzoxazoles [19,20,21,22,23]. Owing to a rigid backbone structure, consisting of a series of fused aromatic rings and to the presence of a shape-persistent site of contortion, the hindered chain packing of PIMs results in exceptionally high free volume, organized in a network of interconnected cavities. These materials have shown very high gas permeation rates, while maintaining acceptable selectivity values, and moreover they demonstrated great thermal and chemical stability [18].

Most experimental studies on prospective membrane materials are performed only with pure gases. While those data constitute a valuable benchmark of the materials properties, pure-gas tests are often insufficient to infer how the materials will behave in mixed-gas conditions. Mixed-gas permeation and sorption experiments have shown significant deviations from pure-gas (“ideal”) behavior, both positive and negative [18,21,24,25,26]. In order to properly design a separation operation, it is necessary to characterize the relevant materials properties, namely its permeability and selectivity, as close as possible to the actual operating conditions, which can vary depending on the origin of the gaseous stream to be treated. Consequently, to uncover all the relevant phenomena, a broad experimental campaign, encompassing a wide range of temperatures, pressures and compositions, would be needed.

The transport of small molecules in dense polymeric membranes is described by the solution-diffusion model [27], according to which permeability (P) is the product of the solubility (S) and diffusion coefficients (D):

(1) P=S·D

Whether ultra-high free volume polymers can still be regarded as dense materials is an open question, however, there have been reports of successful modelling studies relying on this hypothesis [28]. Following this description, the selectivity of the polymer (perm-selectivity) $\alpha_{i,j}$, which is equal to the ratio between the permeability of the two gases, contains a solubility-selectivity ($\alpha_{i,j}^{S}$) and a diffusivity-selectivity factor ($\alpha_{i,j}^{D}$):

(2) $$\alpha_{i,j}=\frac{P_{i}}{P_{j}}=\frac{S_{i}}{S_{j}}\cdot \frac{D_{i}}{D_{j}}=\alpha_{i,j}^{S}\cdot \alpha_{i,j}^{D}$$

Solubility-selectivity is expected to provide an important contribution to the overall permselectivity in high free volume glassy polymers, whereas for low and medium free volume polymers, where sieving effects are more important, the diffusivity-selectivity is expected to have a higher weight in the overall permselectivity. It would be interesting to be able to predict the mixed-gas behavior, using at most only pure-gas experimental measurement as input, in order to avoid or reduce the need for the more delicate and time-consuming mixed-gas tests.

The calculation of gas solubility in glassy polymers is customarily performed in the literature using the Dual Mode Sorption (DMS) model [29,30,31,32,33,34,35,36,37,38,39]. This model divides the total sorbed gas into two contributions: the molecules dissolving into the dense portion of the polymer (following Henry’s law), and those saturating the microvoids of the excess free volume that characterizes the glassy state (described by a Langmuir curve). Its simplicity of use and its capability to correlate well the experimental sorption behavior in glassy polymers in most cases are the main reasons behind its success. However, this model does not allow to represent all types of sorption isotherms encountered, such as the sigmoidal shape of the sorption isotherms of alcohols in glassy polymers. There have been studies aimed at overcoming this limitation: for example, by incorporating multilayer sorption theory, a DMS based model capable of representing all the different shapes of sorption isotherms encountered was developed [40].

Another known issue with the use of this model is that the adjustable polymer-penetrant parameters of the DMS model depend on polymer history and operating conditions, thus lacking predictive ability outside their range of derivation, as discussed, for example, by Bondar at al. concerning the pressure range [41].

Alternatively, one can use an Equation of State (EoS) based approach to evaluate gas sorption equilibria. Some models successfully applied to the study of polymeric materials are those based on a Lattice Fluid (LF) representation of substances [42], or on hard sphere chain schemes, like the Statistical Associating Fluid Theory (SAFT) [43]. In the case of glassy polymers, due to their nonequilibrium condition, equilibrium models, such as an EoS, are not applicable. In these cases the Non-Equilibrium Thermodynamics for Glassy Polymers (NET-GP) approach [44] can be used instead. This approach extends equations of state to the nonequilibrium case, providing nonequilibrium expressions for the free energy of the system, by introducing an internal state variable, the polymer density, to describe the out-of-equilibrium degree of the systems. This approach has been successfully applied to the prediction of gas and vapor sorption in a variety of polymeric systems [28,45,46,47] and its capability to represent mixed-gas sorption equilibria in high free volume glassy polymer has been addressed in another work under preparation [48].

Alternatively, atomistic simulations can be employed for the prediction of sorption isotherms. Monte Carlo simulations in the Grand Canonical ensemble [49] can be performed to this aim, thanks to insertion moves that allow the polymeric system to exchange gas particles with an infinite bath until it reaches the equilibrium concentration corresponding to a given value of the chemical potential. Prediction of sorption isotherms can also be performed by post-processing of Molecular Dynamics (MD) or Monte Carlo (MC) trajectories, using the Widom test particle insertion method [50]: the intermolecular interaction energy felt by a molecule inserted in a random position in the polymer phase is related to the excess chemical potential of the penetrant inside the polymer and, in turn, to its solubility coefficient. In dense systems or in presence of large penetrant molecules, the probability of successful insertion moves decreases significantly and therefore the estimate of solubility through Widom insertions becomes less reliable. Other strategies include the use of gradual insertion of the penetrant molecules [51], or the use of particle deletion moves instead of particle insertions (Staged Particle Deletion [52], Direct Particle Deletion [53]). Atomistic simulation techniques have been increasingly applied in recent years to the study of gas transport properties of microporous polymers [54], and of PIMs in particular [55,56,57,58]. Sorption of CO_2_ in PIM-1 was first simulated by Heuchel et al. [59] employing the Gusev-Suter Transition State Theory [60,61]. Even though the simulated solubility coefficients were significantly higher than the experimental ones, their work paved road to the application of molecular modelling techniques to this class of materials. Fang et al. [62,63] applied the Widom test particle insertion method [50] to predict CO_2_ solubility in PIM-1 and their results were in close agreement with the experimental ones. Recently, Kupgan et al. [64] predicted CO_2_ sorption in PIM-1 up to 50 bar, employing a scheme combining Grand Canonical Monte Carlo (GCMC) and Molecular Dynamics simulations devised by Hölck et al. [65], while Frentrup et al. [66] performed Nonequilibium Molecular Dynamics simulations for the direct simulation of He and CO_2_ permeability through a thin membrane of PIM-1, which was in good qualitative agreement with experimental data.

Fewer modelling studies deal with the analysis of mixed gas sorption effects. Recently Rizzuto et al. [67] have coupled GCMC atomistic simulations and Ideal Adsorbed Solution Theory (IAST) [68] to investigate the mixed-gas permeation properties of CO_2_/N_2_ mixtures in Thermally Rearranged polymers. The simulations underestimated pure-gas sorption of both gases, however their results displayed the competitive effects between the gases expected in the case of glassy polymers, which affect greatly the solubility of the less condensable gas in the mixture. Neyertz and Brown [69] performed large-scale MD simulations of air separation with an ultra-thin polyimide membrane surrounded by an explicit gas reservoir, which allowed them to determine gas solubility, diffusivity and O_2_/N_2_ selectivity in multicomponent conditions, comparing favorably with experimental results. For this gas couple, the modelling study predicted a multicomponent solubility-selectivity comparable to the ideal one, that is calculated as the ratio of pure-gas solubility coefficients.

With the development of more accurate potentials, new algorithms for the generation of amorphous polymeric structures and efficient equilibration protocols, the reliability of the predictions yielded by atomistic techniques has drastically improved over the years [54,70]. Moreover, these techniques have the potential to be employed for screening purposes on existing materials, as well as on yet to be synthesized ones, as demonstrated, for instance, by Hart et al. [57] and Larsen et al. [71] for the case of CO_2_/CH_4_ separation with PIMs. However the extremely high computational effort required by atomistic approaches, combined to the system-specificity of several methods, remains a drawback to their application to the study of the separation properties of polymers, even though multiscale strategies, involving systematic coarse-graining and equilibration of high molecular weight models at the coarse-grained level and subsequent back-mapping to the atomistic detail have been implemented successfully to study a variety of properties of polymeric systems with reduced machine-time [72,73,74,75].

The present work is aimed at modelling gas solubility in high free volume glassy polymers both in pure- and mixed-gas conditions using the multicomponent version of the DMS model [76]. In particular, the sorption of CO_2_/CH_4_ mixtures in PTMSP, PIM-1 and TZ-PIM at several compositions and temperatures was studied, using experimental data presented in previous works to validate the results of the calculations [24,25,26,48]. The characterization of mixed-gas sorption is still quite limited and these materials are among the very few for which these experiments were performed. PTMSP, being the most permeable dense polymer [77], is a natural reference point to assess the separation performance of high free volume materials, as is PIM-1, which was the first material of the PIM class to be reported [15]. TZ-PIM constitutes an attempt at improving the selectivity of PIM-1 towards CO_2_ by incorporating more CO_2_-philic groups into its structure, demonstrating that post-polymerization modification techniques with controlled conversion rates represent a viable way of tuning the separation properties of these innovative materials. In this case the nitrile groups were substituted by tetrazole groups [78], but Satilmis et al. [79] showed that it is also possible to reduce them to primary amines, obtaining a material termed amine-PIM-1, with intermediate features between PIM-1 and TZ-PIM.

Lanč et al. [80] recently performed a critical analysis of the difference between gas solubility coefficients determined directly, with sorption experiments, or indirectly, from the time-lag of permeation. They investigated several high free volume glassy polymers, including PTMSP and PIM-1, concluding that the underlying approximation of a linear concentration profile across the membrane, assumed in the time-lag analysis, is a nonnegligible source of error in the indirect determination of S, but it can be mitigated by the calculation of concentration profiles using the thermodynamic Fick’s law instead [81]. The authors also remarked the importance of sorption studies in uncovering fundamental aspect of gas transport in membrane materials.

**Figure 1:**
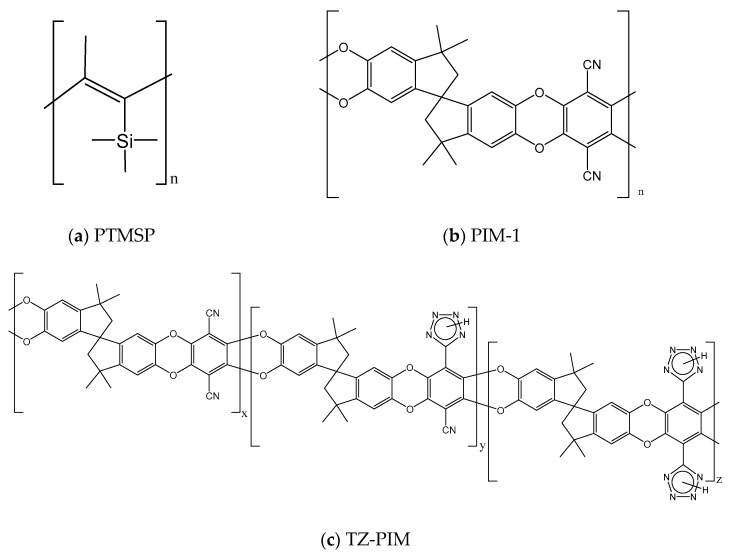
Repeating units of the polymers considered in this study: (**a**) poly(trimethylsilyl propyne) (PTMSP); (**b**) the first reported polymer of intrinsic microporosity (PIM-1); (**c**) tetrazole-modified PIM-1 (TZ-PIM).

Indeed, experimental measurements of mixed-gas sorption [24,25,26,48] allowed to understand that the competition between CO_2_ and CH_4_ plays a strong role in the multicomponent sorption behavior. Furthermore, the data indicate that the pure-gas solubility does not provide a good estimate of the real behavior of the mixture. In particular, pure-gas data would indicate that the main membrane parameters, like the solubility-selectivity, are a strong function of the gas mixture composition, while experimentally it is observed that the data depend very weakly on such variable. Additionally, for a set of glassy polymers comprising poly(2,6-dimethyl-1,4-phenylene oxide) (PPO), PTMSP, PIM-1 and Matrimid^®^, it was shown that departure functions, expressing the deviations between the multicomponent properties and the corresponding ideal values, estimated with pure component properties, obey generalized trends which resemble those observed in liquid solutions [82]. The ability of the Dual Mode Sorption model to represent these physical phenomena, as well as its quantitative accuracy in the prediction of solubility and solubility-selectivity were assessed, by comparing the results of the calculation with experimental data available for the materials considered [24,25,26,48], whose repeating units are shown in Figure 1. Moreover, a sensitivity analysis was carried out, in order to verify the robustness of the calculation and the reliability of the prediction in absence of experimental data for validation.

## 2. Dual Mode Sorption Model

The existence of a sorption mechanism particular to polymers in the glassy state was first postulated by Meares [35,36]. The indication that polymers below the glass transition temperature contain a distribution of microvoids frozen into their structure [35] suggested that those region of reduced density could act as preferential sorption sites. Using this concept and observing that the sorption isotherms of organic vapors in ethyl cellulose showed a curvature concave to the pressure axis, that was not witnessed in the case of rubbery materials, and, furthermore, that for these systems rather high negative values of the heat of solution were measured, Barrer et al. [37] proposed the existence of two concurrent mechanisms of sorption: dissolution and “hole-filling”.

Therefore, the Dual Mode Sorption (DMS) model [29,30,31,32,33,34,35,36,37,38,39] postulates the existence of two different gas populations inside glassy polymers, at equilibrium with one another. One is dissolved in the dense portion of the material and can be described by Henry’s law, while the other saturates the nonequilibrium excess free volume of the polymer and is described by a Langmuir curve. In this schematization, the total sorbed gas as a function of gas fugacity can be expressed as a sum of these two contributions [31,37]:

(3) $$c_{i}=k_{D,i}f_{i}+\frac{C_{H,i}^{\prime }b_{i}f_{i}}{1+b_{i}f_{i}}$$

The parameter $k_{D,i}$ is Henry’s law constant, while $b_{i}$ is Langmuir affinity constant, which represents the ratio of the rate constants of sorption and desorption of penetrants in the microvoids and, therefore, it quantifies the tendency of a given penetrant to sorb according to the Langmuir mode.$C_{H,i}^{\prime }$ is the Langmuir capacity constant, which characterizes the sorption capacity of a glassy polymer for a given penetrant in the low-pressure region. This latter parameter correlates with changes in polymer density associated with formation history or annealing treatments [83,84] and has been shown to disappear at the glass transition temperature (*T_g_*) of the polymer [85]. For every gas-polymer pair and temperature analyzed, the three parameters are retrieved through a nonlinear least-square best fit of pure-gas sorption data.

The extension to multicomponent sorption of this model [76] is based on phenomenological arguments, suggested by the theory of competitive sorption of gases on catalysts, which exhibit a Langmuir behavior: since the amount of unrelaxed free volume in a polymer is fixed and limited (swelling is not taken into account by the model), the various penetrants will compete to occupy it and, as a consequence, the sorbed concentration is expected to decrease with respect to the pure-gas case.

It is assumed that the extent of the competitive effect is controlled by the relative values of the product of the affinity constant and partial pressure (or fugacity) of each penetrant. Under the hypothesis that the affinity parameter b, Henry’s constant $k_{D}$ and the molar density of a component sorbed inside the Langmuir sites are independent of the presence of other penetrants, the expression for the concentration (*c*) of component *i* in presence of a second component *j* is given by Equation (4):

(4) $$c_{i}=k_{D,i}f_{i}+\frac{C_{H,i}^{\prime }b_{i}f_{i}}{1+b_{i}f_{i}+b_{j}f_{j}}$$

The characteristic gas-polymer parameters of the model are retrieved at each temperature from a least-square fit of a pure-gas isotherm using Equation (3). These parameters are subsequently used to predict the concentration of each gas in mixed-gas conditions at several compositions, making use of Equation (4) [86]. Therefore, in Equation (4), all parameters are the same as found in Equation (3).

It is also commonplace to write Equations (3) and (4) using the partial pressure of each gas instead of its fugacity. When high pressures are considered, such as in the present study, the approximation of ideal-gas behavior is not valid. Therefore, the fugacity is generally considered instead of partial pressure, since it constitutes a more appropriate measure of the gas chemical potential, which is the driving force for gas sorption in the polymer. Moreover, when mixtures are concerned, two gases like CH_4_ and CO_2_ show different departures from ideality, meaning that they can have the same partial pressure, but rather different fugacity. Even though the accuracy of the pure-gas data representation with the DMS model using either variable is the essentially the same, the values of the parameters obtained using pressure or fugacity are clearly different [87], therefore it should always be specified which variable was used in the regression, in order to enable a meaningful comparison between different parameter sets. In the context of mixed-gas sorption measurements, results are more often reported using gas fugacity, to account for the different degree of non-ideality of the components in the gas phase, therefore this was the natural choice of variable for this study as well. The accuracy of the results does not depend on this choice: it was verified that using pressure-based parameters or fugacity-based parameters yielded the same results in the mixed-gas sorption calculations. The same observation was reported also by Sanders et al. [87,88] in their studies on mixed-gas sorption of binary mixtures in poly(methyl methacrylate) (PMMA), and by Story et al. [89], in their work on mixed-gas sorption in PPO. They found that, the use of pressure-based or fugacity-based DMS parameters in the calculation of mixed-gas sorption yielded very similar results, only slightly more accurate in some of the cases when fugacity was used instead of partial pressure.

The evaluation of the solubility-selectivity is also of interest (Equation (5)). This performance indicator can be calculated using the solubility coefficients of the pure gases, as it is often done when mixture data are not available (ideal case), or, more accurately, using the solubility coefficients obtained in mixed-gas sorption tests/calculations (multicomponent case).

(5) $$\alpha_{CO_{2}/CH_{4}}^{S}=\frac{S_{CO_{2}}}{S_{CH_{4}}}=\frac{c_{CO_{2}}/f_{CO_{2}}}{c_{CH_{4}}/f_{CH_{4}}}$$

Since a fugacity-based representation was adopted, the solubility coefficients (S) are defined as the ratio of gas concentration (*c*) and gas fugacity (f). The fugacity of the gases at various pressures was calculated with the Peng-Robinson equation of state (EoS) [90], both in pure- and mixed-gas condition evaluations. In the mixed-gas case, the binary parameter $k_{CO_{2}/CH_{4}}=0.09$ [91] has been used in the Peng-Robinson EoS.

The DMS model does not account for the fact that the polymer matrix, unlike rigid porous materials, can swell when sorbing penetrants. Therefore, possible synergistic effects due to second-component induced swelling are not accounted for by such approach. However, ultra-high free volume glassy polymers have a limited tendency to swell, and the experimental data collected so far on mixed-gas sorption of CO_2_/CH_4_ mixtures indicate that such effects are not predominant in these materials, at least for pressures of CO_2_ below 30 bar. On the other hand, in these conditions, it was observed that the prevailing multicomponent effect is the one associated with competition during sorption. Therefore, in the cases examined here, the DMS model is expected to provide a reliable estimation of the data [87].

## 3. Results

### 3.1. Pure-Gas Sorption Analysis

DMS parameters for the materials analyzed, obtained from a least-square fitting procedure with the Generalized Reduced Gradient (GRG) method [92], using concentration vs. fugacity data, are reported in Table 1. At each temperature, a different parameter set was obtained, and no constraints were applied to enforce a temperature dependence. In the last columns of the table, the Standard Error of the Estimate (*SEE*) is reported, as goodness-of-fit indicator. *SEE* was used instead of the correlation coefficient (*R*^2^), because the underlying assumptions in the definition of *R*^2^ are not valid in the case of a nonlinear regression model, such as the DMS model [93,94,95]. The following definition was used in its calculation:

(6) $$SEE\equiv \sqrt{\frac{\sum_{i}{\left(y_{i,exp}-y_{i,calc}\right)}^{2}}{n-p}}$$

In the definition of *SEE* (Equation (6)), $y_{i,exp}$ are the experimental points, $y_{i,calc}$ are the corresponding values calculated with the model, *n* is the number of experimental points used in the regression and *p* is the number of parameters employed by the model. *SEE* is expressed in concentration units (as *y*) and lower values indicate a better agreement between experimental and calculated values. For the mixed-gas prediction, the reported value $\bar{SEE}$*_mix_* is the average deviation from three sorption isotherms at different composition calculated with the same pure-gas parameter sets.

**Figure 2:**
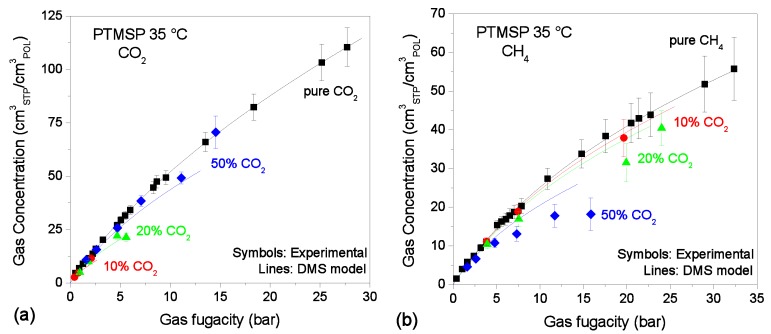
Sorption isotherms of CO_2_ (**a**) and CH_4_ (**b**) at 35 °C in PTMSP, in pure- and mixed-gas conditions (Black squares: pure-gas; Red circles: 10% CO_2_ mixture; Green triangles: 20% CO_2_ mixture; Blue diamonds: 50% CO_2_ mixture). Experimental data from [24]. Solid lines represent Dual Mode Sorption (DMS) model predictions.

**Figure 3:**
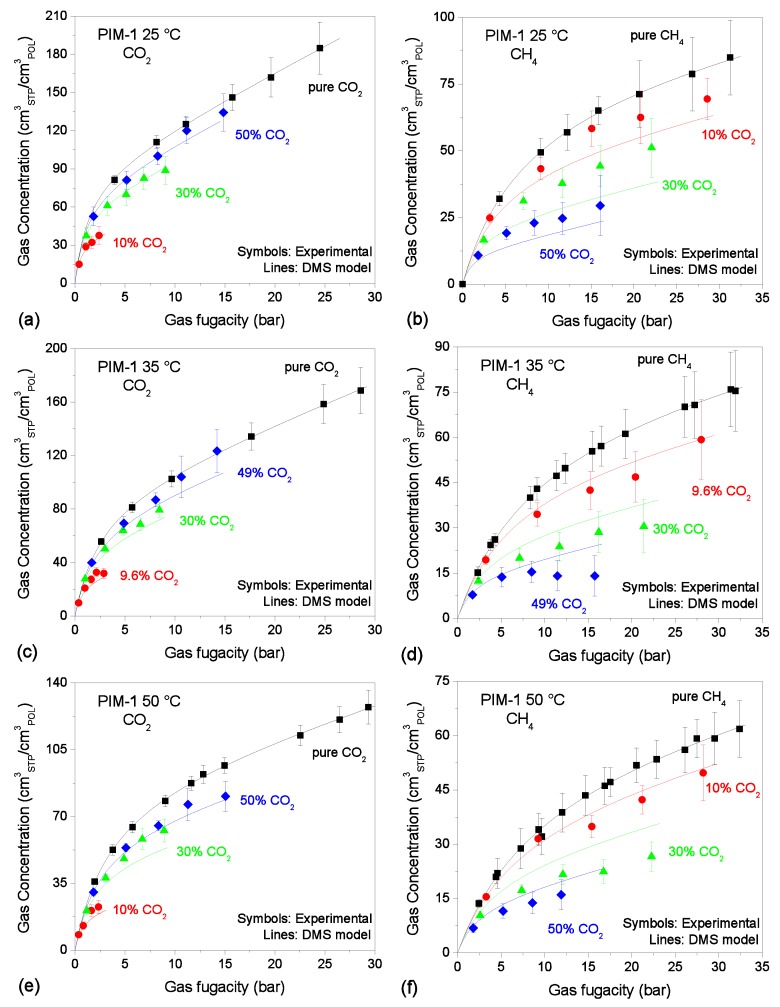
Sorption isotherms of CO_2_ and CH_4_ at 25 °C (**a**,**b**); 35 °C (**c**,**d**); 50 °C (**e**,**f**) in PIM-1, in pure- and mixed-gas conditions (Black squares: pure-gas; Red circles: ~10% CO_2_ mixture; Green triangles: ~30% CO_2_ mixture; Blue diamonds: ~50% CO_2_ mixture). Experimental data from [26]. Solid lines are DMS model predictions.

**Figure 4:**
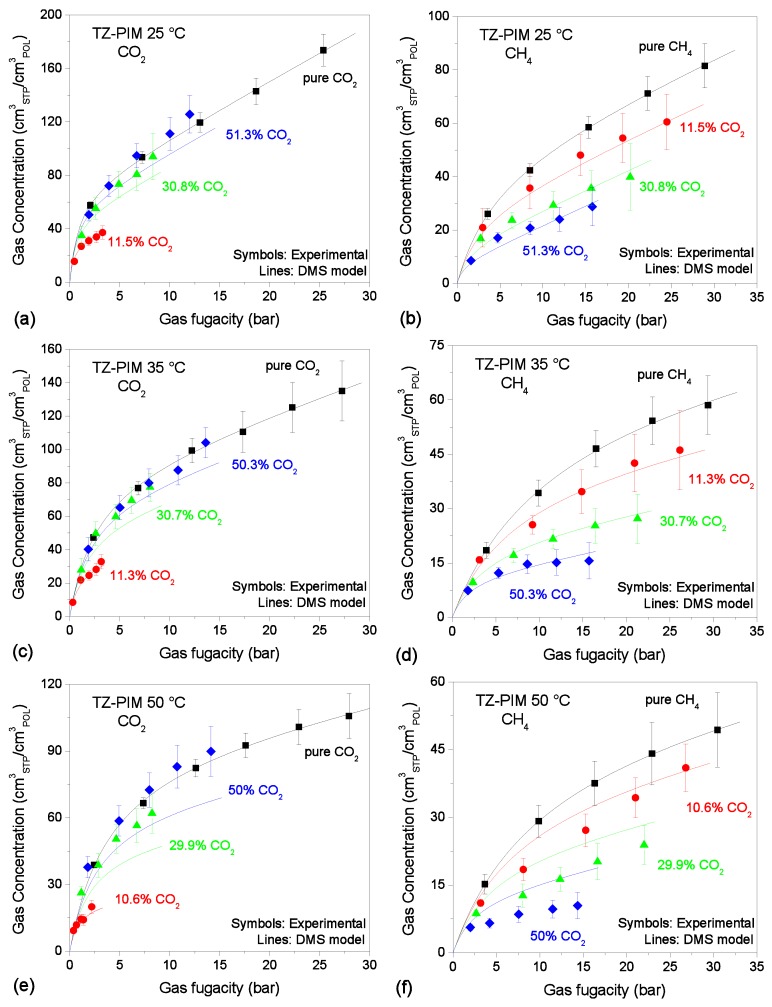
Sorption isotherms of CO_2_ and CH_4_ at 25 °C (**a**,**b**); 35 °C (**c**,**d**); 50 °C (**e**,**f**) in TZ-PIM, in pure and mixed-gas conditions (Black squares: pure gas; Red circles: ~10% CO_2_ mixture; Green triangles: ~30% CO_2_ mixture; Blue diamonds: ~50% CO_2_ mixture). Experimental data from [48]. Solid lines are DMS model predictions.

The results are shown in Figure 2, Figure 3 and Figure 4. The model provides an excellent fit to all the pure-gas experimental data sets. Typically, more condensable penetrants, like CO_2_ in the present case, exhibit larger affinity constants, and this was indeed observed in the parameters retrieved. In addition, it would be expected that the presence of the tetrazole CO_2_-philic groups in TZ-PIM would translate into higher affinity constants for CO_2_ sorption, compared to PIM-1. However, this correlation of the parameter with the chemistry of the materials was not observed at all three temperatures, but only in the parameter set for the 25 °C case. This issue might relate to the parametrization route adopted, and it will be further discussed in Section 4.1.

Generally, $k_{D}$*,* $C_{H}^{\prime }$ and b are expected to decrease as temperature increases [76,96,97], consistently with their physical meaning. In the case of $k_{D}$ and b, this trend was verified in all the cases inspected here, while for $C_{H}^{\prime }$ the expected trend was observed only in one case (CH_4_ in PIM-1), while in the other cases the values fluctuated more. If the regression at each temperature is performed independently, fluctuations of the parameters have to be expected. This was noted also by Stevens et al. [98] in their analysis of Dual Mode Sorption model parameters for CO_2_, CH_4_ and N_2_ in HAB-6FDA polyimide and its Thermally Rearranged analogues: when an unconstrained regression was performed independently at each temperature, the expected trends were followed only in some of the cases considered. In order to obtain a consistent parameter set, they imposed temperature dependence during the regression. The effect of these constraints on the mixed-gas sorption prediction will be examined in Section 4.1.

It has been reported that the DMS parameters are sensitive to the pressure range over which they are regressed [41], in particular b tends to decrease and $C_{H}^{\prime }$ to increase, if a broader regression range is considered, and, therefore, extrapolation outside the derivation range should be avoided. In this study, the whole isotherms were used in the regression and the pressure range was the same (0–35 bar) in all cases considered.

### 3.2. Mixed-Gas Sorption: PTMSP

Figure 2 shows the experimental sorption data of CO_2_/CH_4_ mixtures (10/20/50 mol.% CO_2_) in PTMSP at 35 °C [24] together with the results of mixed-gas sorption calculations with the DMS parameters reported in Table 1.

The predictions are in very good agreement with the experimental data in the case of CO_2_, while in the case of CH_4_ at high pressure the model overestimates the concentration for the 30:70 and 50:50 mixtures, with a maximum relative deviation of 20% and 35% respectively. Nonetheless, the model captures the fact that there is competition between the gases during sorption, but also that it is less pronounced in this polymer than in the other materials analyzed here, even at high values of the fugacity of the second component.

### 3.3. Mixed-Gas Sorption: PIM-1

Figure 3 shows the experimental sorption data of CO_2_/CH_4_ mixtures (~10/30/50 mol.% CO_2_) in PIM-1 at 25, 35, 50 °C [25,26], together with the results of mixed-gas sorption calculations with the DMS model. It can be seen that, in the case of CO_2_, the prediction is accurate at the lowest temperature, with average relative deviations below 5%. The average relative deviations, however, are increased to 10% at 35 °C and 50 °C.

On the other hand, in the case of CH_4_, the accuracy is lower and its trend with temperature is opposite with respect to the case of CO_2_. At 25 °C the concentration is significantly underestimated at all compositions (the average relative deviation is 19%), while at 35 °C it is overestimated by a similar extent (the average relative deviations is 18%). At 50 °C CH_4_ sorption is still overestimated by the model, but the prediction is slightly more satisfactory, with average relative deviations of 15%. The deviation between the experimental data and the model predictions is greater than the experimental confidence intervals in several cases, therefore it does not seem to be explained fully by the uncertainty in the mixed-gas sorption measurements. Generally, for all temperatures analyzed, the lowest deviations are seen for both gases in the mixture case in which they are more abundant (50% CO_2_ and 90% CH_4_ respectively).

Not much can be done *a priori* to improve the quantitative accuracy of the mixed-gas prediction, because the parametrization at each temperature is independent and relies only on the accuracy of the pure-gas sorption measurements. However the effect of using a different parametrization route will be discussed in a later section.

### 3.4. Mixed-Gas Sorption: TZ-PIM

In Figure 4, the experimental sorption data of CO_2_/CH_4_ mixtures (~10/30/50 mol.% CO_2_) in TZ-PIM at 25, 35, 50 °C [48], together with the results of mixed-gas sorption calculations with the DMS model are reported. In the case of TZ-PIM, the prediction of CO_2_ sorption is more accurate at 25 °C and 35 °C (10% average relative deviations), while it worsens at 50 °C, where the model would seem to underestimate CO_2_ concentration both in the 30% CO_2_ and in the 50% CO_2_ mixtures, with average relative deviations with respect to the experimental data of 26% and 23%, which are greater than the experimental confidence intervals.

In the case of CH_4_, at 25 °C and 35 °C DMS predictions show very good agreement with the experimental data, with average relative deviations below 5% at all compositions. Conversely, at 50 °C the model significantly overestimates the CH_4_ concentration, by as much as 32% on average.

### 3.5. Solubility-selectivity

Ideal and multicomponent solubility-selectivities were evaluated using Equation (5). At a fixed value of the total pressure of the mixed-gas feed, the corresponding fugacity of each gas in the mixture and multicomponent concentration values were used to obtain the multicomponent solubility-selectivity. The ideal solubility-selectivity was evaluated using the same fugacity values as in the multicomponent case, but with the corresponding concentration values taken from the pure-gas sorption isotherms.

**Figure 5:**
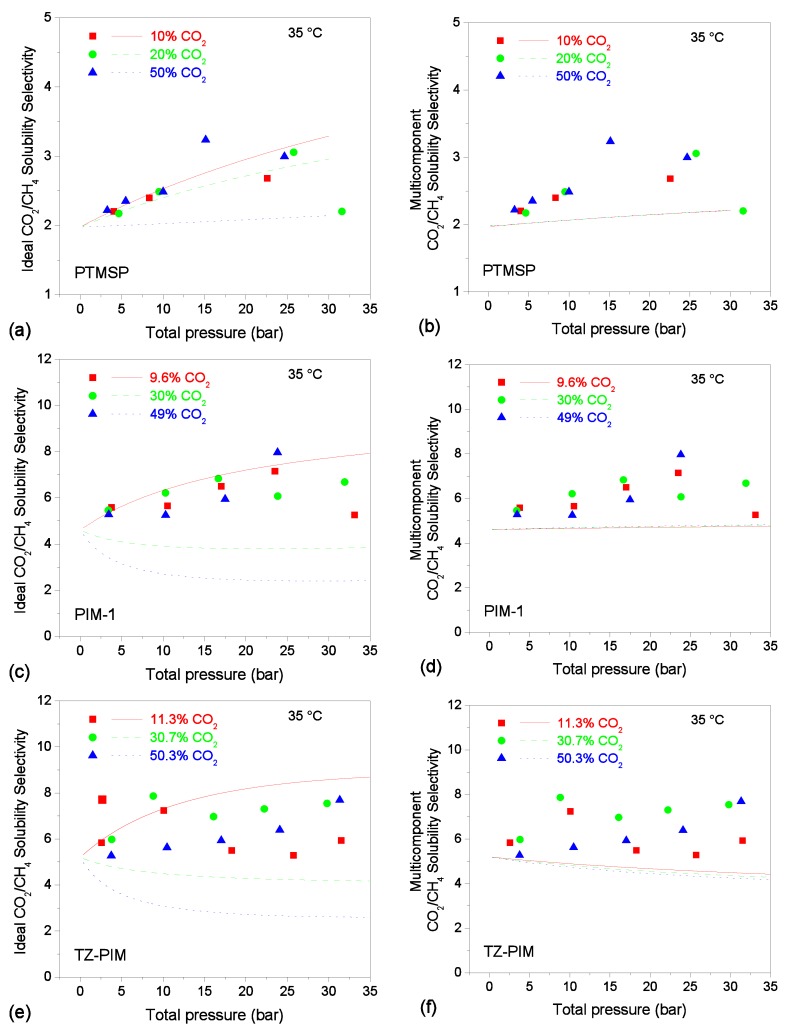
CO_2_/CH_4_ solubility-selectivity in PTMSP, PIM-1, and TZ-PIM at 35 °C at various mixture compositions. Points are experimental values [24,25,48], lines represent calculations with the DMS model. In the left column, i.e., (**a**,**c**,**e**), the lines represent ideal solubility-selectivity values, calculated with pure-gas concentrations. In the right column, i.e., (**b**,**d**,**f**), the lines represent the calculated multicomponent solubility-selectivity values.

The obtained trends are compared with the experimental data in Figure 5. For the sake of brevity, results of the comparison are shown only for the 35 °C case, but all the general observations that follow were true also for the results at 25 °C and 50 °C.

A common remark to all cases inspected is that the mixed-gas calculations show a very different dependence on mixture composition and total pressure with respect to the ideal case calculation. In particular, mixed-gas calculations generally show a much weaker dependence than the ideal ones versus both pressure and composition. It seems, therefore, that the competitive effect, accounted for in the mixed-gas calculations, tends to stabilize the calculated solubility-selectivity with respect to fluctuations in the gas pressure and composition. The physical reason beyond this behavior, that is also confirmed by experiments, is not completely clear.

In particular, for PTMSP, the calculated values are close to the experimental ones and the trends predicted by the model exhibit almost no dependence on the gas mixture composition, with the three curves collapsing onto one another, whereas the experimental data are more scattered, and resemble more the results of the ideal-case calculation, in the lower CO_2_ content cases (10–20% CO_2_) and low pressure-range, where indeed the gas phase is closer to and ideal one.

Similarly, the gas composition dependence of solubility-selectivity is negligible in the mixed-gas calculation for PIM-1, and there is almost no dependence on pressure as well. In the case of TZ-PIM, the calculated values in the mixed-gas case show a very modest concentration and pressure dependence, although slightly more marked than in the other cases.

The calculated values for PIM-1 and TZ-PIM slightly underestimate the solubility-selectivity, but they would be preferable than simpler ideal-case estimates (left column of Figure 5), which, on average, could lead to larger errors. Indeed, in the evaluation of the selectivity, the experimental error of both gas concentrations is combined and, therefore, this parameter inevitably has a higher uncertainty. For this reason, it is not straightforward to infer pressure and gas mixture composition dependencies from the experimental data, due to large fluctuations and absence of monotonous trends. Nonetheless, it is clear that the calculations performed with mixed-gas concentrations yield significantly more accurate results than using the corresponding pure-gas values.

## 4. Sensitivity Analysis

### 4.1. Pure-Gas Sorption Isotherms Fitting Method

Reasons for the deviation of the DMS model predictions from the experimental data were identified originally by Koros [76] in the possible presence of non-negligible penetrant-penetrant specific interactions, or as consequence of swelling and plasticization effects, which are not accounted for in the model and that would make the parameters concentration-dependent, or require the introduction of additional terms and adjustable parameters. There have been extensions and modifications to the DMS model to include this aspects with the introduction of additional parameters [40,99,100] but the original version is still the most used one.

Since the DMS model parametrization is carried out independently at each temperature, it is striking that the accuracy of the mixed-gas prediction reported in Figure 3 and Figure 4 varies so much between different temperatures. In order to address this issue, a different parametrization route was tried and, subsequently, a sensitivity analysis of the mixed-gas calculation to the parameter set was performed.

New parameter sets were obtained (Table 2) by taking into account during the nonlinear least-square optimization the experimental error associated with each experimental point, by minimizing $\chi^{2}$ defined as follows [94]:

(7) $$\chi^{2}=\overset{N}{\underset{i=1}{\sum }}\frac{1}{\sigma_{i}^{2}}{\left[c_{i}-\left(k_{D,i}f_{i}+\frac{C_{H,i}^{\prime }b_{i}f_{i}}{1+b_{i}f_{i}}\right)\right]}^{2}$$

here $\sigma_{i}$ represents the confidence interval associated with the experimental value of the concentration $c_{i}$, N is the total number of experimental points, $f_{i}$ is the gas fugacity and $k_{D,i}$, $C_{H,i}^{\prime }$, $b_{i}$ are the DMS parameters for the polymer-*i* penetrant couple. Since very often only data at one reference temperature are available, each data set was treated independently, without additional constraints to impose a temperature dependence to the parameters.

As expected, slightly different parameter sets from the ones reported in Table 1 were obtained. It was observed that an increase in the value of $C_{H,i}^{\prime }$ was always accompanied by a decrease in the values of $k_{D,i}$ and $b_{i}$, and vice versa. In the electronic supplementary information (ESI) file, the comparison of the mixed-gas predictions obtained with the parameter sets from Table 1 and Table 2 is reported in Figures S1–S3, where also the experimental confidence intervals are included for reference. It is remarkable that pure-gas representations are almost indistinguishable (as it can be noted also by the very similar values of Standard Error associated to the two parameter sets), even though some of the values of the parameters used differ by as much as 30%.

The mixed-gas calculations performed with the parameters reported in Table 2 provided a modest improvement in the accuracy of the prediction in some of the cases analyzed (CO_2_ in TZ-PIM at 35 and 50 °C, CH_4_ in TZ-PIM at 50 °C, CO_2_ in PIM-1 at 35 °C, CH_4_ in PIM-1 at 25 and 35 °C, CH_4_ in PTMSP at 35 °C), whereas in other cases they produced slightly less accurate results (CO_2_ in TZ-PIM at 25 °C, CH_4_ in TZ-PIM at 25 and 35 °C, CO_2_ in PIM-1 at 25 and 50 °C, CH_4_ in PIM-1 at 50 °C, CO_2_ in PTMSP at 35 °C). A systematic trend was not detected, at times the average accuracy was increased for both gases at the same temperature (TZ-PIM at 50 °C case and PIM-1 at 35 °C case), other times only for one of the two gases at the same temperature (TZ-PIM at 35 °C case, PIM-1 25 °C case and PTMSP 35 °C case) and in other cases for none (TZ-PIM 25 °C case and PIM-1 50 °C case). Moreover, in some instances, the results were more accurate at certain compositions but worse at others. On the whole, the discrepancies between the accuracy of the multicomponent calculations in different conditions were not eliminated by taking into account the experimental error during the parametrization.

To improve the internal consistency of the parameters, a multi-temperature parametrization scheme was tested. For each gas, new parameters were obtained by considering the data at all temperatures simultaneously and constraining the parameters to follow the expected temperature dependence. In particular, the temperature dependence of $k_{D}$ and b is described by a van’t Hoff relation [96]:

(8) $$k_{D}=k_{D0}\;e^{-\frac{\Delta H_{D}}{RT}}$$

(9) $$b=b_{0\;}e^{-\frac{\Delta H_{b}}{RT}}$$

In Equations (8) and (9) $\Delta H_{D}$ and $\Delta H_{b}$ are the enthalpies of sorption for Henry and Langmuir modes, R is the gas constant and T is the temperature. The pre-exponential factors $k_{D0}$ and $b_{0}$, together with $\Delta H_{D}$ and $\Delta H_{b}$ were treated as adjustable coefficients. For $C_{H}^{\prime }$, no functional temperature dependence was imposed, but the values were constrained to diminish with increasing temperature. In this way, to obtain the parameters for each gas-polymer couple at three temperatures, only 7 adjustable coefficients were used, instead of 9.

The parameter sets obtained are reported in Table 3. It is noteworthy that, in this parameter set, the values of Langmuir affinity constant for the couple CO_2_-PIM-1 are always lower than the corresponding ones for CO_2_-TZ-PIM at each temperature, as it would be expected given the chemical difference between the two materials.

The comparison of the mixed-gas predictions obtained with the parameter sets from Table 1 and Table 3 is reported in Figures S4 and S5 in the ESI file. To satisfy the temperature dependence constraint, a slightly less accurate representation of pure-gas sorption is generally observed, especially at higher pressure. The largest deviations are observed in the cases of CO_2_ and CH_4_ sorption in TZ-PIM at 25 and 50 °C, for CO_2_ sorption in PIM-1 at 35 °C and for CH_4_ sorption in PIM-1 at 25 °C.

The mixed-gas sorption calculations performed with the parameters reported in Table 3 provided a slight improvement in the accuracy of the prediction of CO_2_ and CH_4_ sorption in PIM-1 at all temperatures. On the other hand, in the case of TZ-PIM, the mixed-gas calculation yielded comparable results for both gases at 50 °C, but less accurate predictions for both gases in the 35 °C case. At 25 °C, the results for CH_4_ sorption in TZ-PIM were slightly better compared to those obtained with the parameter set without a consistent temperature dependence, while those for CO_2_ were slightly worse. Therefore, on the whole, this parametrization route presented improvements, in terms of internal consistency of the parameter set and their physical interpretation, but it still didn’t eliminate the variability in the accuracy of the mixed-gas sorption results.

In order to address the issue systematically, a sensitivity analysis was carried out: the effect of CO_2_ and CH_4_ parametrization on the multicomponent calculation was studied separately, analyzing how the mixed-gas sorption results are affected by variations of $C_{H,i}^{\prime }$, $k_{D,i}$, $b_{i}$ while $b_{j}$ is kept fixed, and, subsequently, the effect of changes in the value of $b_{j}$ were also taken into account.

### 4.2. Confidence Intervals of the DMS Model Parameters

A comprehensive search in the parameters space was conducted, using a grid method, in order to identify a range of DMS model parameter values that allow to obtain equally satisfactory representations of the pure-gas data. Once such a range was estimated, it was tested whether different parameter sets, within those confidence intervals, could lead to better mixed-gas predictions than the ones obtained with the best-fit sets.

The results are presented in the following for the case of CH_4_ solubility in PIM-1. Results analogous to the ones presented in this section were obtained also for the other cases. They are not shown, for the sake of brevity, but they can be found in the ESI file (Figures S6 and S7 complete the analysis of CH_4_ sorption in PIM-1 at 35 °C and 50 °C, Figures S8–S12 and Tables S1 and S2 show the results for the case of CO_2_ sorption in PIM-1 and the outcome of the calculations for CO_2_ and CH_4_ sorption in TZ-PIM are reported in Tables S3 and S4 and in Figures S13–S20).

**Figure 6:**
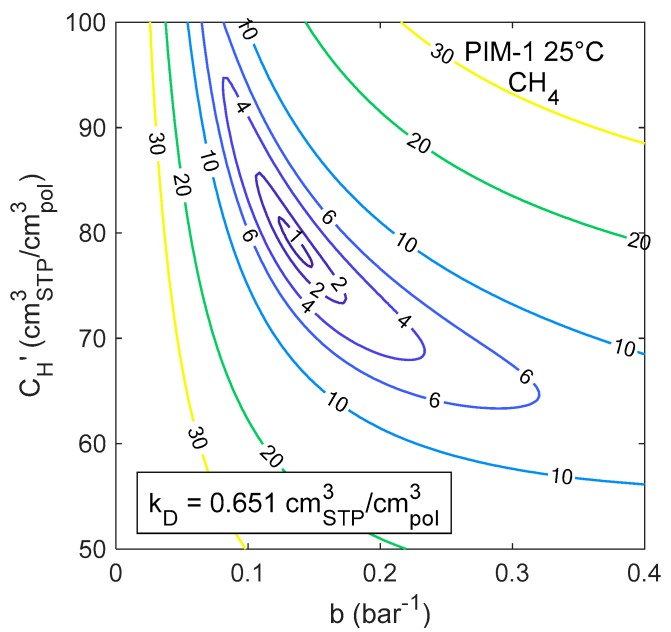
Contour plot of the Standard Error of the Estimate (SEE) of CH_4_ sorption in PIM-1 at 25 °C, obtained varying the Langmuir sorption parameters, with a fixed value of the Henry’s constant.

Figure 6 shows a contour plot of the Standard Error in the calculation of pure CH_4_ sorption in PIM-1 at 25 °C, obtained varying b and $C_{H}^{\prime }$ while holding $k_{D}$ constant at its best fit value: 0.651 cm^3^_STP_/cm^3^_pol_. Each line represents a locus of constant *SEE* in the Langmuir parameter space, for a fixed value of Henry’s constant.

**Figure 7:**
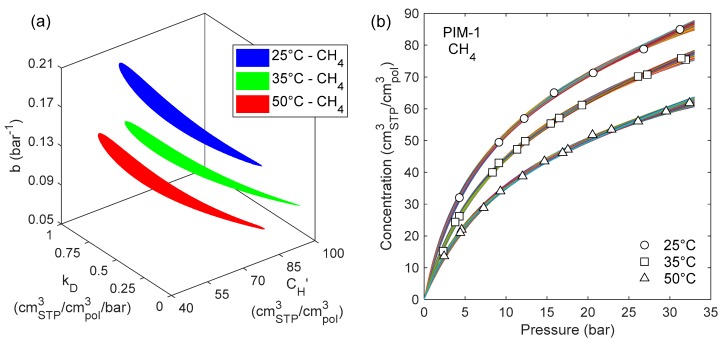
(**a**) Surfaces enclosing the range where DMS parameter sets yield *SEE < SEE_max_* in the prediction of CH_4_ sorption in PIM-1 at three different temperatures; (**b**) CH_4_ sorption isotherms in PIM-1 at 25, 35 and 50 °C, calculated with all the parameter sets enclosed by the corresponding colored regions in the plot on the left.

When $k_{D}$ is allowed to vary as well, surfaces at constant error in the three-dimensional parameter space are obtained. As a criterion to delimit the confidence intervals, a maximum value for the standard error was selected. This value was chosen as *SEE_max_* = 1 cm^3^_STP_/cm^3^_pol_ at 25 °C, which corresponds to an average relative deviation of 1.5%. The same relative deviation is attained with *SEE_max_* = 0.9 cm^3^_STP_/cm^3^_pol_ at 35 °C and *SEE_max_* = 0.8 cm^3^_STP_/cm^3^_pol_ at 50 °C. In Figure 7a a 3D plot is presented, in which the three colored regions correspond to domains in the parameter space where *SEE* < *SEE_max_* for CH_4_ sorption in PIM-1 at the three different temperatures. Therefore, each point within the colored region corresponds to a parameter set that satisfies the accuracy criterion. The sorption isotherms obtained with all the parameter sets included in the colored regions of part (a) of the figure are represented together in Figure 7b, and one can see that there is indeed a small, but detectable, variation in the representation of the experimental data using either of the parameter sets. This variability, however, is always lower than the experimental uncertainty of the data.

The upper and lower limits in each direction of the isosurfaces reported in Figure 7a can be used to attribute confidence intervals to the DMS parameters. Confidence intervals for nonlinear regression functions are often asymmetrical and this was observed also by other authors for the DMS model parameters [21,98].

Confidence intervals of the DMS parameters for CH_4_ sorption in PIM-1 are reported in Table 4. Clearly, not all the combinations of parameters within their respective confidence interval would give a valid set, otherwise the confidence region in the 3D parameter space would be represented by a parallelepiped. However, for all values included in the confidence interval of one parameter, it would be possible to find values of the other two parameters such that the accuracy criterion is satisfied. As a consequence, when using only one of the parameters, like in the case of $b_{j}$ in Equation (4), all values belonging to its confidence interval should be considered acceptable.

### 4.3. Evaluation of Mixed-Gas Sorption

All the parameter sets that satisfied the condition *SEE* < *SEE_max_* in the pure-gas sorption representation, were used to calculate mixed-gas sorption isotherms, using the best-fit values reported in Table 1 for $b_{CO_{2}}$.

**Figure 8:**
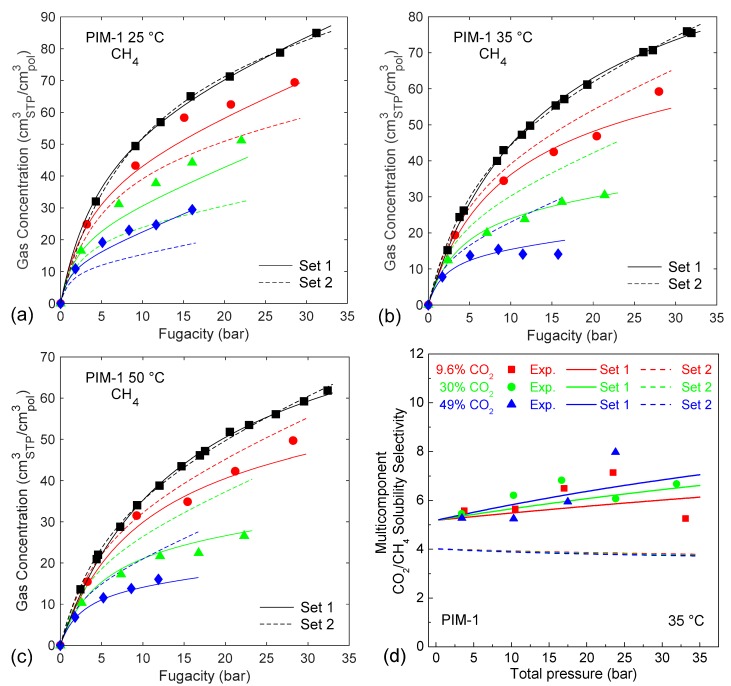
Dual Mode Sorption model mixed-gas predictions of CH_4_ sorption in PIM-1 at 25 °C (**a**); 35 °C (**b**); 50 °C (**c**) obtained with the two parameter sets reported in Table 5; In (**d**) the solubility-selectivity calculated with the two sets at 35 °C is compared. Solid lines are obtained with Set 1, dashed ones with Set 2.

To quantify the accuracy of the mixed-gas prediction (${\bar{SEE}}_{mix}$), the average *SEE* of isotherms at three concentrations (10/30/50 mol.% CO_2_) for each temperature was used, and then the lowest and the highest results were selected, in order to identify the best and worst predictions, labelled respectively Set 1 and Set 2. The parameter sets that correspond to these two extreme cases and their *SEE* values are summarized in Table 5. The calculated sorption isotherms for each case are shown in Figure 8.

At each temperature, both parameter sets have a remarkably similar accuracy in the representation of pure-gas data, but they yield a significantly different prediction of the mixed-gas behavior. For instance, the solid lines in Figure 8 (obtained with Set 1) show a very good agreement with the experimental data, whereas the dashed ones (obtained with Set 2) are even less accurate than the initial result obtained with the best-fit parameter set.

Allowing also for experimental error, the two pure-gas representations at each temperature (black lines in Figure 8) are deemed to be equivalent and no reason for choosing one over the other could be suggested. Therefore, in absence of mixed-gas experimental data to validate against, the confidence in the accuracy of the calculation would be weakened.

The same variability was observed also in the case of CH_4_ sorption in TZ-PIM, while for CO_2_ sorption in both PIM-1 and TZ-PIM, the uncertainty in the mixed-gas predictions was generally lower (see Figures S9 and S14 in the ESI file).

This is reflected also in the evaluation of the solubility-selectivity: comparing the results obtained with Set 1 and Set 2 (Figure 8d) it is possible to see that not only the results obtained with Set 1 are in much better agreement with the experimental data, but also that the pressure and concentration dependence predicted by the two sets is significantly different, and in one case it is consistent with the general trend of the experimental points, while in the other case it is the opposite.

**Figure 9:**
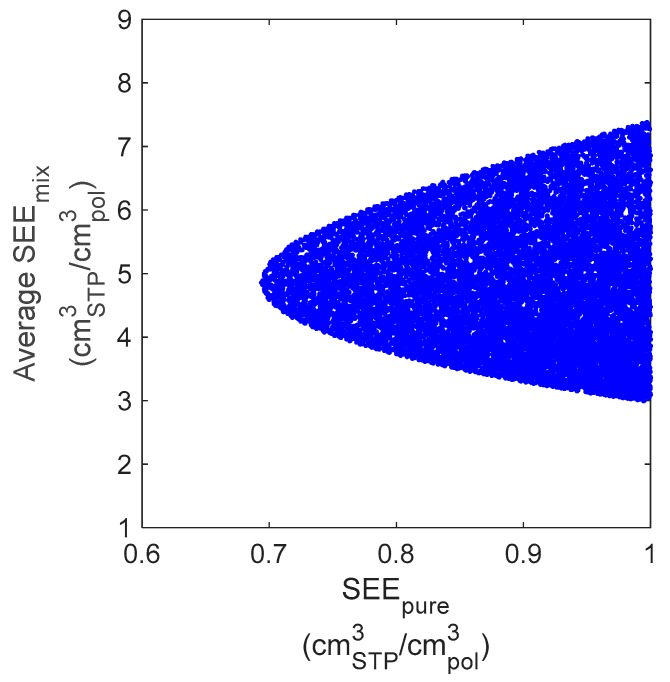
Accuracy range of the mixed-gas prediction (*y*-axis) for CH_4_ in PIM-1 at 25 °C corresponding to a given accuracy in the pure-gas data representation (*x*-axis). Each point represents the result obtained with a different parameter set among those enclosed in the colored region of Figure 7a.

By looking at the relationship between the deviations in the pure-gas data fitting and the mixed-gas prediction, the level of uncertainty that is inherent in the calculation becomes more apparent. In Figure 9 the deviation in the pure-gas data representation ($SEE_{pure}$) is related to the error in the mixed-gas prediction (${\bar{SEE}}_{mix}$). Each point in the plot represents a calculation performed with a different parameter set, among those meeting the accuracy criterion (Figure 7a).

It can be seen that, when moving to the right, i.e., further away from the best-fit parameter set and therefore towards slightly higher deviations in the pure-gas data correlation, a small variation of $SEE_{pure}$ results in a much wider range of possible outcomes for ${\bar{SEE}}_{mix}$, thus the reliability of the calculation becomes less predictable. The interval of the values assumed by ${\bar{SEE}}_{mix}$ expands both towards higher and lower values, so that better mixed-gas predictions can indeed be found with slightly “less accurate” pure-gas data representations, and not with the best-fit parameter set.

### 4.4. Effect of $b_{CO_{2}}$

The mixed-gas sorption results depend also strongly on the value of the Langmuir affinity constant of the second component ($b_{CO_{2}}$), which is found in the Dual Mode Sorption model expression for sorbed concentration in the multicomponent case. Following the same procedure adopted for CH_4_, the region in the DMS parameter space of CO_2_ sorption in PIM-1 at 25 °C satisfying the condition *SEE* < *SEE_max_* was identified. It is reported in Figure S6 of the ESI file, alongside confidence regions at 35 °C and 50 °C. From this analysis, it was possible to identify the confidence interval over which $b_{CO_{2}}$ could vary $\left(b_{CO_{2}}^{25\;{}^{\circ}C}=0.710_{\;-0.239}^{\;+0.272}\right)$ and then study the influence of its variations on the CH_4_ mixed-gas sorption prediction.

**Figure 10:**
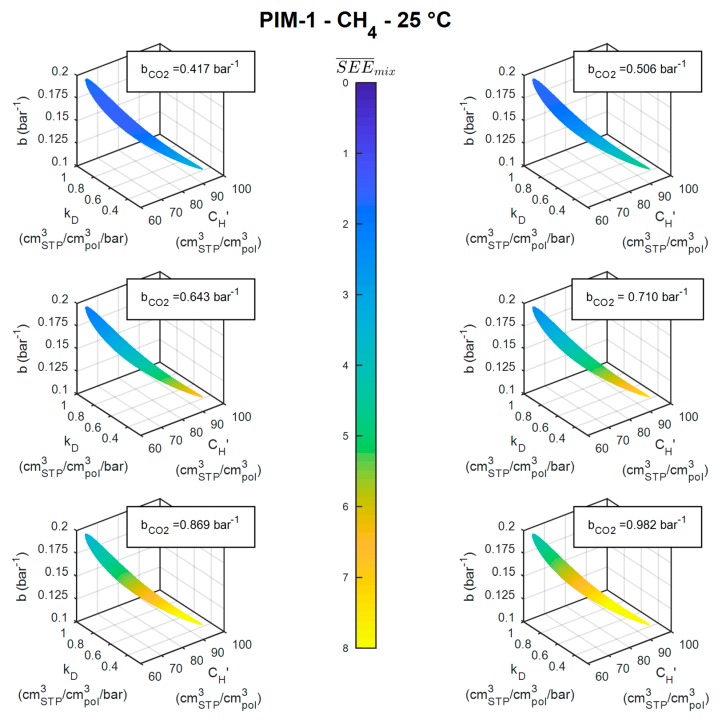
Isosurfaces in the DMS parameter space of CH_4_ sorption in PIM-1 at 25 °C corresponding to SEE_pure_ < SEE_max_, colored according to the average SEE_mix_ obtained with different values within the confidence interval of $b_{CO_{2}}$.

In Figure 10, the same region of acceptable parameter sets for CH_4_ sorption in PIM-1 at 25 °C found in Figure 7a is represented, but, in these plots, a color scale indicates the average accuracy in the mixed-gas prediction corresponding to each point in the plot. The calculation is repeated for different values of $b_{CO_{2}}$, chosen to span its entire *SEE* < *SEE_max_* region. It can be seen that a greater accuracy of the mixed-gas predictions is not attained with the best-fit value of $b_{CO_{2}}$ reported in Table 1 (0.710 bar^−1^), but with a lower one instead (top-left corner of Figure 10), whereas with higher values it becomes increasingly difficult to have good predictions at all (bottom-right corner of Figure 10). Once more, this was not a general trend. For example, in the cases of CH_4_ sorption in PIM-1 at 35 and 50 °C, the more accurate mixed-gas predictions could be attained with values of $b_{CO_{2}}$ higher than the best fit one, as it can be observed in Figures S6 and S7 of the ESI file.

## 5. Discussion

Due to the form of Equations (3) and (4), the parameters $C_{H}^{\prime }$ and b are strongly coupled and, therefore, a deviation of either of them can be compensated by a corresponding deviation of the other, yielding a similar overall quality of the fit. The same remark was made also by Gleason et al. [21] in their analysis of Dual Mode parameters for mixed-gas permeation of CO_2_/CH_4_ in Thermally Rearranged HAB-6FDA. In order to improve the accuracy of the calculation, they chose to incorporate mixed-gas data into the fitting procedure used to retrieve the DMS parameters. Raharjo et al. [97] studied sorption of CH_4_-*n*C_4_H_10_ mixtures in PTMSP and they noticed a tendency of the DMS model to systematically overestimate CH_4_ concentration in mixed-gas conditions. They subsequently re-parametrized the model, including the mixture data as well, obtaining different parameter sets from those retrieved considering only pure-gas data. In both cases [21,97] the representation of the mixture behavior was superior when the multicomponent data was included during the parametrization, but the procedure is clearly no longer predictive.

In order to reduce the uncertainty in the regression of the DMS parameters, Wang et al. [101] suggested to obtain Henry’s constant independently, through the analysis of the temperature dependence of the solubility coefficient above *T_g_*, and then retrieve only $C_{H}^{\prime }$ and b from the best-fit of the sorption data. This approach yielded different sets from those obtained in a simultaneous regression of all three parameters and, even though those sets had lower values of the goodness-of-fit indicator, they showed improved self-consistency and the expected temperature dependence. This method, however, was not applicable to the materials studied here, and in general for glassy polymers with very high *T_g_*, for which gas solubility data above *T_g_* are not available.

Comparing the results displayed here for mixed-gas CH_4_ sorption in PIM-1 to those of CH_4_ sorption in TZ-PIM and also to those of CO_2_ sorption in PIM-1 and TZ-PIM, it is not straightforward to identify a general trend and therefore draw guidelines to mitigate the issue. The parameter set obtained by imposing a temperature dependence yielded the most reliable results, therefore, this parametrization route should be followed whenever possible, if the intended use of the parameters is that of performing predictive mixed-gas sorption calculations. If data at only one temperature are available and the quantitative accuracy of the mixed-gas sorption is necessitated, caution in the use of this model is advised.

In general, the prediction was either satisfactory for all compositions or for none: a low average *SEE_mix_* was always the consequence of a similar representation of all three mixed-gas sorption isotherms. Therefore, if one could validate the parameter set adopted at least against experimental data at one composition, it should be possible to calculate the behavior at other compositions with greater confidence.

## 6. Conclusions

The sorption of CO_2_/CH_4_ mixtures in three high free volume glassy polymers, PTMSP, PIM-1 and TZ-PIM, was modelled with the multicomponent extension of the Dual Mode Sorption model. The three model parameters were retrieved from the best fit of pure-gas sorption isotherms and yielded an excellent representation of the experimental data. Multicomponent calculations provided a good qualitative picture of sorption in mixed-gas conditions, displaying the reduction in the solubility that is observed experimentally, due to competition with the second gas present in the mixture, and how this effect is more pronounced for the less soluble gas (CH_4_). Moreover, a reasonable estimate of solubility-selectivity was obtained. The quantitative agreement with the experimental data in multicomponent conditions, however, varied greatly between the cases inspected.

A sensitivity analysis revealed that a small uncertainty in the pure-gas data, which would translate into a different parametrization, is greatly amplified in the mixed-gas calculation, which is much less robust than expected. Great variability is also introduced by the affinity constant of the second component, $b_{j}$, which could also assume slightly different values due to the experimental uncertainty. In the absence of experimental mixed-gas data to validate against, it is difficult to estimate a priori the quantitative accuracy of the mixed-gas prediction.

In conclusion, given its simplicity and immediacy of use, this model is a useful tool for a first estimate of the mixed-gas effects, but great care should be used when quantitative accuracy is of interest, resorting eventually to other, yet more complex, models instead [48].

## Figures and Tables

**Table 1:** Dual Mode Sorption model parameters (fugacity-based) for CO_2_ and CH_4_ sorption in PTMSP, PIM-1 and TZ-PIM, obtained by a least-square fit on data from Refs. [24,25,26,48] to Equation (3).

**CO_2_**						
	***T***	$k_{D}$	$C_{H}^{\prime }$	b	***SEE***	$\bar{SEE}$ ***_mix_***
	**(°C)**	$\left(\frac{cm_{STP}^{3}}{cm_{pol}^{3}bar}\right)$	$\left(\frac{cm_{STP}^{3}}{cm_{pol}^{3}}\right)$	**(** $bar^{-1})$	$\left(\frac{cm_{STP}^{3}}{cm_{pol}^{3}}\right)$	$\left(\frac{cm_{STP}^{3}}{cm_{pol}^{3}bar}\right)$
PTMSP	35	1.973	95.06	0.051	0.67	0.94
PIM-1	25	4.046	90.04	0.710	1.38	2.06
	35	2.890	94.83	0.388	0.92	3.90
	50	1.596	89.30	0.290	0.80	2.08
TZ-PIM	25	4.127	70.58	1.127	1.28	9.77
	35	1.982	89.53	0.378	1.57	6.66
	50	0.903	92.42	0.263	1.02	11.00
**CH_4_**						
	***T***	$k_{D}$	$C_{H}^{\prime }$	b	***SEE***	$\bar{SEE}$ ***_mix_***
	**(°C)**	$\left(\frac{cm_{STP}^{3}}{cm_{pol}^{3}bar}\right)$	$\left(\frac{cm_{STP}^{3}}{cm_{pol}^{3}}\right)$	**(** $bar^{-1})$	$\left(\frac{cm_{STP}^{3}}{cm_{pol}^{3}}\right)$	$\left(\frac{cm_{STP}^{3}}{cm_{pol}^{3}bar}\right)$
PTMSP	35	0.616	57.77	0.049	0.50	1.78
PIM-1	25	0.651	78.83	0.136	0.70	4.89
	35	0.541	75.87	0.106	0.51	2.69
	50	0.543	57.90	0.105	0.89	1.70
TZ-PIM	25	1.400	48.09	0.214	0.55	2.54
	35	0.378	67.12	0.087	0.97	1.40
	50	0.350	51.41	0.101	0.20	4.73

**Table 2:** Dual Mode Sorption model parameters (fugacity-based) for CO_2_ and CH_4_ sorption in PTMSP, PIM-1, TZ-PIM, obtained by a least-square fit on data from Refs. [24,25,26,48] to Equation (7).

**CO_2_**						
	***T***	$k_{D}$	$C_{H}^{\prime }$	b	***SEE***	$\bar{SEE}$ ***_mix_***
	**(°C)**	$\left(\frac{cm_{STP}^{3}}{cm_{pol}^{3}bar}\right)$	$\left(\frac{cm_{STP}^{3}}{cm_{pol}^{3}}\right)$	**(** $bar^{-1})$	$\left(\frac{cm_{STP}^{3}}{cm_{pol}^{3}}\right)$	$\left(\frac{cm_{STP}^{3}}{cm_{pol}^{3}bar}\right)$
PTMSP	35	2.373	69.26	0.067	0.73	0.97
PIM-1	25	3.664	100.25	0.506	1.60	3.72
	35	3.039	90.42	0.428	1.17	3.43
	50	1.666	86.84	0.306	0.84	2.57
TZ-PIM	25	4.023	72.88	1.019	1.44	11.21
	35	2.168	84.61	0.420	1.82	6.16
	50	1.150	84.84	0.303	1.46	10.11
**CH_4_**						
	***T***	$k_{D}$	$C_{H}^{\prime }$	b	***SEE***	$\bar{SEE}$ ***_mix_***
	**(°C)**	$\left(\frac{cm_{STP}^{3}}{cm_{pol}^{3}bar}\right)$	$\left(\frac{cm_{STP}^{3}}{cm_{pol}^{3}}\right)$	**(** $bar^{-1})$	$\left(\frac{cm_{STP}^{3}}{cm_{pol}^{3}}\right)$	$\left(\frac{cm_{STP}^{3}}{cm_{pol}^{3}bar}\right)$
PTMSP	35	0.611	58.75	0.048	0.52	0.92
PIM-1	25	0.672	78.56	0.137	0.72	3.00
	35	0.401	82.72	0.097	0.60	1.68
	50	0.684	50.99	0.124	0.94	2.08
TZ-PIM	25	1.526	43.65	0.250	0.76	2.97
	35	0.282	73.42	0.079	0.98	1.61
	50	0.364	50.63	0.103	0.20	4.18

**Table 3:** Dual Mode Sorption model parameters (fugacity-based) for CO_2_ and CH_4_ sorption in PIM-1, TZ-PIM, obtained by a least-square fit on data from Refs. [25,26,48], imposing the temperature dependence constraints expressed by Equations (8) and (9).

**CO_2_**						
	***T***	$k_{D}$	$C_{H}^{\prime }$	b	***SEE***	$\bar{SEE}$ ***_mix_***
	**(°C)**	$\left(\frac{cm_{STP}^{3}}{cm_{pol}^{3}bar}\right)$	$\left(\frac{cm_{STP}^{3}}{cm_{pol}^{3}}\right)$	**(** $bar^{-1})$	$\left(\frac{cm_{STP}^{3}}{cm_{pol}^{3}}\right)$	$\left(\frac{cm_{STP}^{3}}{cm_{pol}^{3}bar}\right)$
PIM-1	25	4.150	90.56	0.638	2.43	1.86
	35	2.883	89.02	0.470	4.30	3.73
	50	1.742	85.63	0.308	1.11	1.44
TZ-PIM	25	3.625	82.25	0.676	3.54	13.43
	35	2.473	77.23	0.511	3.13	10.04
	50	1.457	76.94	0.347	2.92	11.31
**CH_4_**						
	***T***	$k_{D}$	$C_{H}^{\prime }$	b	***SEE***	$\bar{SEE}$ ***_mix_***
	**(°C)**	$\left(\frac{cm_{STP}^{3}}{cm_{pol}^{3}bar}\right)$	$\left(\frac{cm_{STP}^{3}}{cm_{pol}^{3}}\right)$	**(** $bar^{-1})$	$\left(\frac{cm_{STP}^{3}}{cm_{pol}^{3}}\right)$	$\left(\frac{cm_{STP}^{3}}{cm_{pol}^{3}bar}\right)$
PIM-1	25	0.759	78.51	0.130	1.80	3.74
	35	0.561	72.98	0.113	0.73	2.02
	50	0.369	66.17	0.093	1.00	1.12
TZ-PIM	25	1.275	49.29	0.206	2.73	2.41
	35	0.865	42.49	0.171	1.99	3.87
	50	0.506	41.58	0.133	1.23	4.46

**Table 4:** Confidence intervals of the fugacity-based DMS parameters yielding and average relative deviation < 1.5% in the calculation of CH_4_ sorption in PIM-1 at three different temperatures.

*T*	$k_{D,CH_{4}}$	$C_{H,CH_{4}}^{\prime }$	$b_{CH_{4}}$
(°C)	$\left(\frac{cm_{STP}^{3}}{cm_{pol}^{3}bar}\right)$	$\left(\frac{cm_{STP}^{3}}{cm_{pol}^{3}}\right)$	($bar^{-1})$
25	$0.651_{\;-0.290}^{\;+0.336}$	$78.83_{\;-15.28}^{\;+15.67}$	$0.136_{\;-0.031}^{\;+0.059}$
35	$0.541_{\;-0.408}^{\;+0.316}$	$75.87_{\;-15.93}^{\;+23.78}$	$0.106_{\;-0.029}^{\;+0.042}$
50	$0.543_{\;-0.443}^{\;+0.237}$	$57.90_{\;-12.74}^{\;+25.60}$	$0.105_{\;-0.035}^{\;+0.049}$

**Table 5:** DMS model fugacity-based parameter sets used in the calculation of mixed-gas sorption of CH_4_ in PIM-1 reported in Figure 8.

	*T*	$k_{D,CH_{4}}$	$C_{H,CH_{4}}^{\prime }$	$b_{CH_{4}}$	*SEE_pure_*	$\bar{SEE}$ *_mix_*
	(°C)	$\left(\frac{cm_{STP}^{3}}{cm_{pol}^{3}bar}\right)$	$\left(\frac{cm_{STP}^{3}}{cm_{pol}^{3}}\right)$	$\left(bar^{-1}\right)$	$\left(\frac{cm_{STP}^{3}}{cm_{pol}^{3}bar}\right)$	$\left(\frac{cm_{STP}^{3}}{cm_{pol}^{3}bar}\right)$
*Set 1*	25	0.945	65.08	0.186	0.999	3.98
	35	0.119	101.81	0.074	0.891	1.15
	50	0.125	82.00	0.070	0.797	1.35
*Set 2*	25	0.316	96.99	0.102	0.996	11.16
	35	0.869	59.26	0.152	0.899	4.95
	50	0.767	45.62	0.155	0.792	3.67

## Supplementary Materials

An Electronic Supplementary Information file is available online at http://www.mdpi.com/2077-0375/9/1/8/s1, containing additional Figures and Tables. Figure S1: Effect of including the experimental error during the DMS model parametrization on the prediction of mixed-gas sorption of CO_2_ and CH_4_ in PTMSP, Figure S2: Effect of including the experimental error during the DMS model parametrization on the prediction of mixed-gas sorption of CO_2_ and CH_4_ in PIM-1, Figure S3: Effect of including the experimental error during the DMS model parametrization on the prediction of mixed-gas sorption of CO_2_ and CH_4_ in TZ-PIM, Figure S4: Effect of constraining the temperature dependence of the parameters during the DMS model parametrization on the prediction of mixed-gas sorption of CO_2_ and CH_4_ in PIM-1, Figure S5: Effect of constraining the temperature dependence of the parameters during the DMS model parametrization on the prediction of mixed-gas sorption of CO_2_ and CH_4_ in TZ-PIM, Figure S6: Effect of $b_{CO_{2}}$ on the calculated mixed-gas sorption of CH_4_ in PIM-1 at 35 °C, Figure S7: Effect of $b_{CO_{2}}$ on the calculated mixed-gas sorption of CH_4_ in PIM-1 at 50 °C, Figure S8: Confidence intervals of CO_2_/PIM-1 DMS model parameters, Figure S9: Range of variation of the DMS model predictions of CO_2_ sorption in PIM-1 in multicomponent conditions, Figure S10: Effect of $b_{CH_{4}}$ on the calculated mixed-gas sorption of CO_2_ in PIM-1 at 25 °C, Figure S11: Effect of $b_{CH_{4}}$ on the calculated mixed-gas sorption of CO_2_ in PIM-1 at 35 °C, Figure S12: Effect of $b_{CH_{4}}$ on the calculated mixed-gas sorption of CO_2_ in PIM-1 at 50 °C, Figure S13: Confidence intervals of CO_2_/TZ-PIM and CH_4_/TZ-PIM DMS model parameters, Figure S14: Range of variation of the DMS model predictions of CO_2_ and CH_4_ sorption in TZ-PIM in multicomponent conditions, Figure S15: Effect of $b_{CO_{2}}$ on the calculated mixed gas sorption of CH_4_ in TZ-PIM at 25 °C, Figure S16: Effect of $b_{CO_{2}}$ on the calculated mixed-gas sorption of CH_4_ in TZ-PIM at 35 °C, Figure S17: Effect of $b_{CO_{2}}$ on the calculated mixed-gas sorption of CH_4_ in TZ-PIM at 50 °C, Figure S18: Effect of $b_{CH_{4}}$ on the calculated mixed-gas sorption of CO_2_ in TZ-PIM at 25 °C, Figure S19: Effect of $b_{CH_{4}}$ on the calculated mixed gas sorption of CO_2_ in TZ-PIM at 35 °C, Figure S20: Effect of $b_{CH_{4}}$ on the calculated mixed-gas sorption of CO_2_ in TZ-PIM at 50 °C, Table S1: Confidence intervals of CO_2_/PIM-1 DMS model parameters, Table S2: DMS parameter sets yielding the most and least accurate predictions of CO_2_ sorption in PIM-1 in multicomponent conditions, Table S3: Confidence intervals of CO_2_/TZ-PIM and CH_4_/TZ-PIM DMS model parameters, Table S4: DMS parameter sets yielding the most and least accurate predictions of CO_2_ and CH_4_ sorption in TZ-PIM in multicomponent conditions.
