# Supplementary material for: Modelling Mixed-Gas Sorption in Glassy Polymers for CO2 Removal: A Sensitivity Analysis of the Dual Mode Sorption Model
Source: Membranes (Basel). 2019 Jan 4;9(1):8. doi: 10.3390/membranes9010008 (PMC6359057; doi:10.3390/membranes9010008)
Supplement: Supplementary file 1 [file membranes-09-00008-s001.pdf]

# Supplementary Materials: Modelling Mixed-Gas Sorption in Glassy Polymers for $CO_2$ Removal: A Sensitivity Analysis of the Dual Mode Sorption Model

**Eleonora Ricci , Maria Grazia De Angelis**

---

## 1. Mixed-Gas Sorption Predictions with Dual Mode Sorption Model Best Fit Parameter Sets Obtained Through Different Parametrization Routes

### *1.1. Error-Weighted Sum of Squared Errors*

Figures S1 to S3 show the comparison between mixed-gas sorption of $CO_2$ and $CH_4$ in PTMSP, PIM-1 and TZ-PIM calculated with the Dual Mode Sorption (DMS) model using two different best-fit parameter sets. Solid lines in the figures are obtained with best-fit parameters resulting from the minimization of the sum of squared errors, weighted using the experimental confidence intervals:

$$\chi^2 = \sum_{i=1}^{N} \frac{1}{\sigma_i^2}\left[c_i - \left(k_{D,i} f_i + \frac{C'_{H,i} b_i f_i}{1 + b_i f_i}\right)\right]^2 \qquad \text{(S1)}$$

$\sigma_i$ represents the confidence interval associated with the experimental value of the concentration $c_i$, $N$ is the total number of experimental points, $f_i$ is the gas fugacity and $k_{D,i}$, $C'_{H,i}$, $b_i$ are the DMS parameters for the polymer-$i$ penetrant couple.

Dashed lines in the figures are obtained with best-fit parameters resulting from minimizing the sum of squared errors, unweighted:

$$\chi^2 = \sum_{i=1}^{N} \left[c_i - \left(k_{D,i} f_i + \frac{C'_{H,i} b_i f_i}{1 + b_i f_i}\right)\right]^2 \qquad \text{(S2)}$$

The parameter sets obtained using Equation S1 or Equation S2 are reported in Table 1 and Table 2 in the main text, respectively.

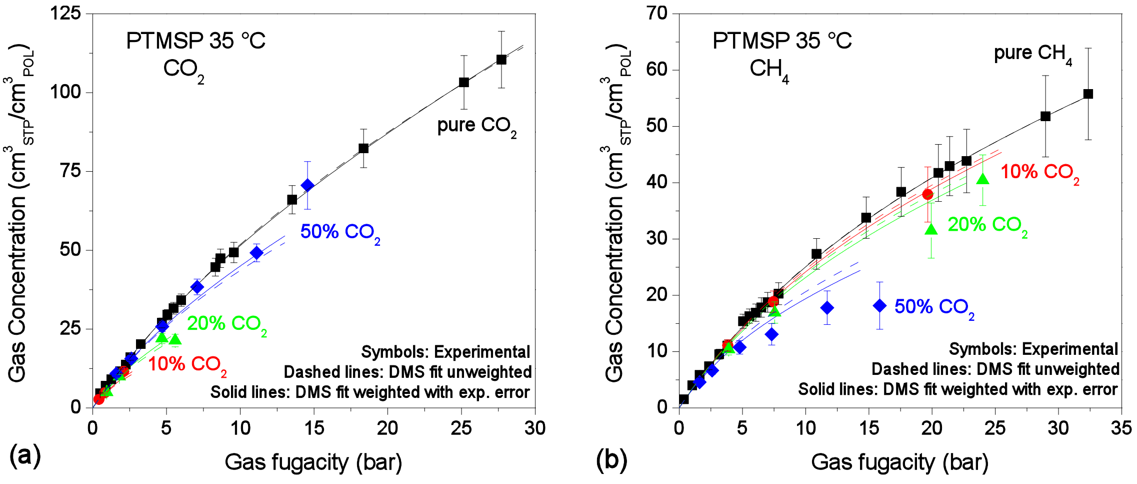


**Figure S1.** Sorption isotherms of (**a**) $CO_2$ and (**b**) $CH_4$ at 35 °C in poly(trimethylsilyl propyne) (PTMSP), in pure and mixed-gas conditions (Black squares: pure gas; Red circles: ~10% $CO_2$ mixture; Green triangles: ~20% $CO_2$ mixture; Blue diamonds: ~50% $CO_2$ mixture). Exp. data from [1]. Solid lines are Dual Mode Sorption (DMS) model predictions obtained using the parameters reported in Table 1 in the main text. Dashed lines are DMS model predictions obtained with parameters reported in Table 2 in the main text.

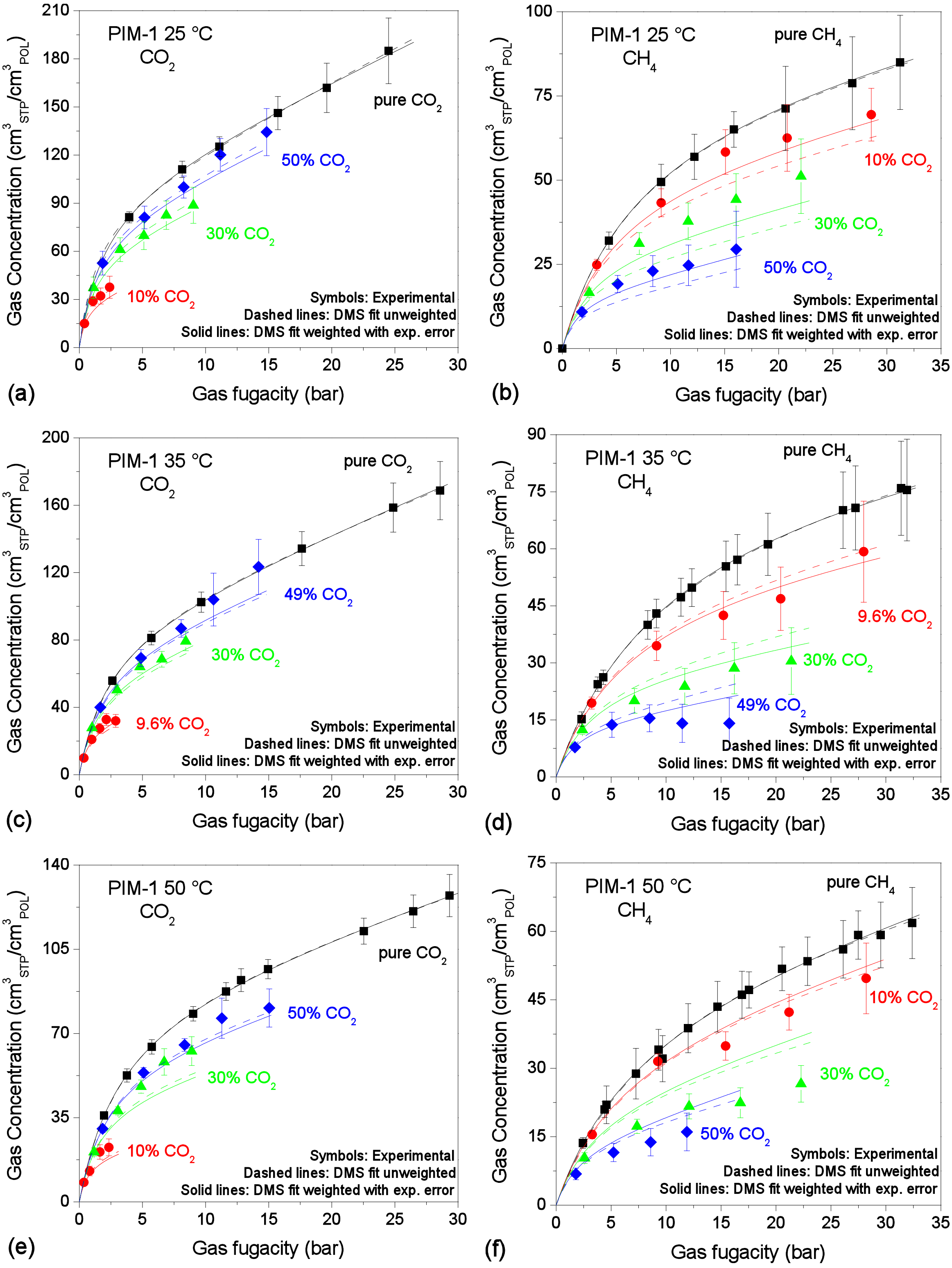


**Figure S2.** Sorption isotherms of $CO_2$ and $CH_4$ at 25 °C (**a**,**b**), 35 °C (**c**,**d**), 50 °C (**e**,**f**) in PIM-1, in pure and mixed-gas conditions (Black squares: pure gas; Red circles: ~10% $CO_2$ mixture; triangles: ~30% $CO_2$ mixture; Blue diamonds: ~50% $CO_2$ mixture). Experimental data from [2,3]. Solid lines represent DMS model predictions obtained using the parameters reported in Table 1 in the main text. Dashed lines are DMS model predictions obtained with parameters reported in Table 2 in the main text.

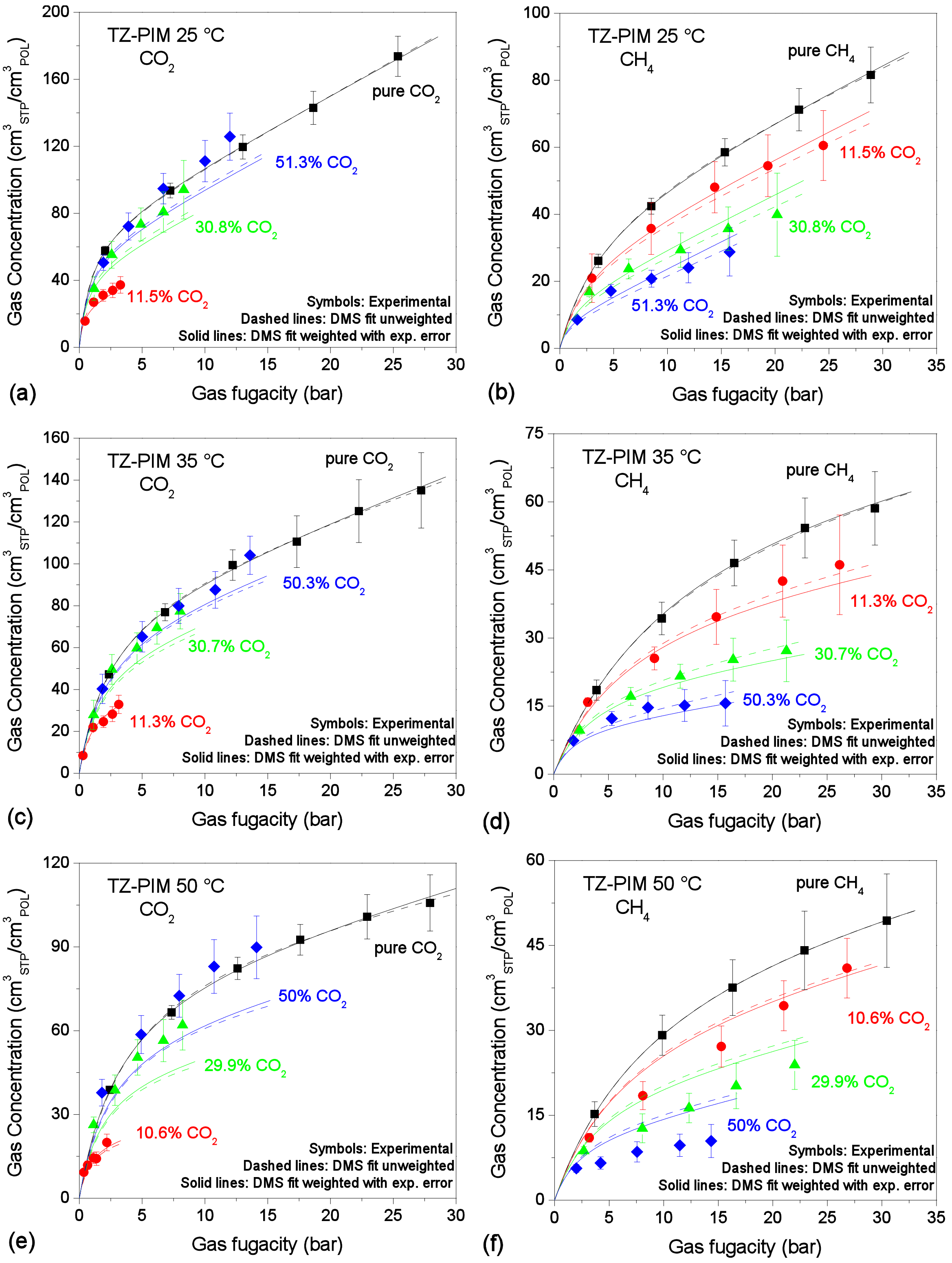


**Figure S3.** Sorption isotherms of $CO_2$ and $CH_4$ at 25 °C (**a**,**b**), 35 °C (**c**,**d**), 50 °C (**e**,**f**) in TZ-PIM, in pure and mixed-gas conditions (Black squares: pure gas; Red circles: ~10% $CO_2$ mixture; Green triangles: ~30% $CO_2$ mixture; Blue diamonds: ~50% $CO_2$ mixture). Experimental data from [4]. Solid lines represent DMS model predictions obtained using the parameters reported in Table 1 in the main text. Dashed lines are DMS model predictions obtained with parameters reported in Table 2 in the main text.

*1.2. Constrained Temperature Dependence*

Figures S4 and S5 show the comparison between mixed-gas sorption of $CO_2$ and $CH_4$ in PIM-1 and TZ-PIM calculated with the Dual Mode Sorption (DMS) model using two different best-fit parameter sets.

Dashed lines in the figures are obtained with best-fit parameters reported in Table 1 in the main text, resulting from minimizing the sum of squared errors, unweighted (Equation S2).

Solid lines in the figures are obtained with best-fit parameters reported in Table 2 in the main text, resulting from the simultaneous minimization of the sum of squared errors at three temperatures, imposing a van't Hof temperature dependence to $b$ and $k_D$, and constraining $C'_H$ to decrease as temperature increases.

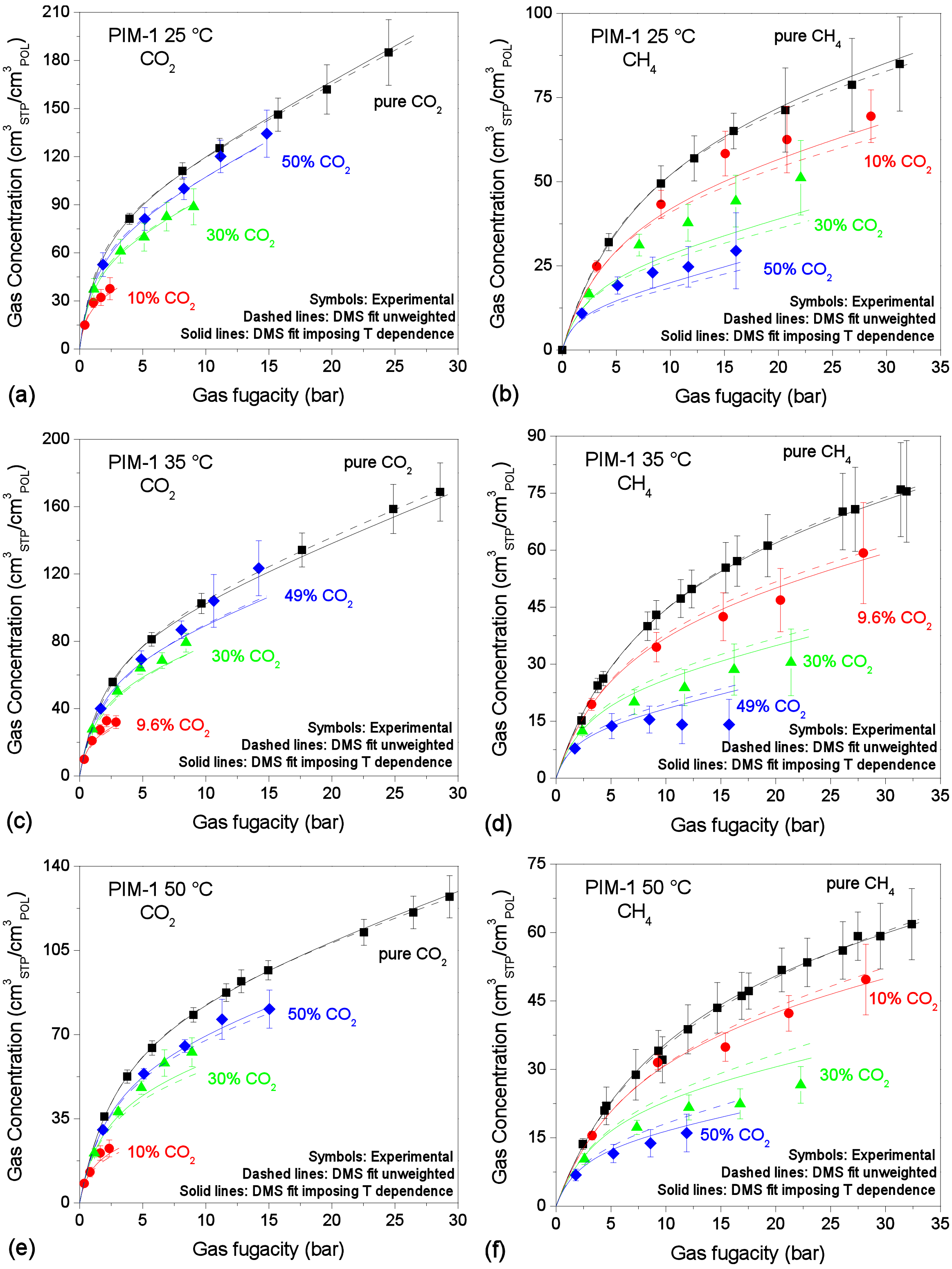


**Figure S4.** Sorption isotherms of $CO_2$ and $CH_4$ at 25 °C (**a**,**b**), 35 °C (**c**,**d**), 50 °C (**e**,**f**) in PIM-1, in pure and mixed-gas conditions (Black squares: pure gas; Red circles: ~10% $CO_2$ mixture; Green triangles: ~30% $CO_2$ mixture; Blue diamonds: ~50% $CO_2$ mixture). Experimental data from [2,3]. Solid lines represent DMS model predictions obtained using the parameters reported in Table 3 in the main text. Dashed lines are DMS model predictions obtained with parameters reported in Table 1 in the main text.

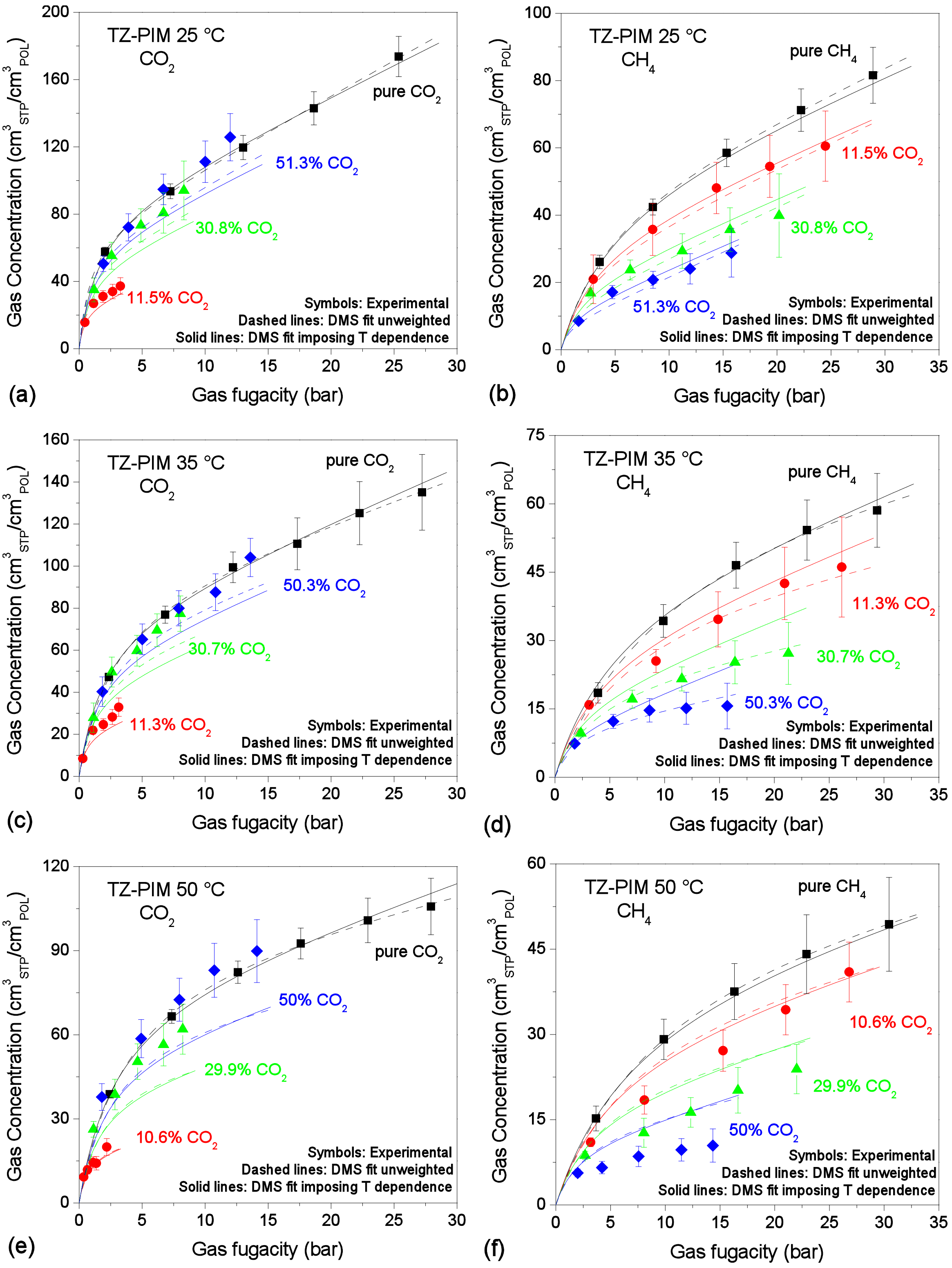


**Figure S5.** Sorption isotherms of $CO_2$ and $CH_4$ at 25 °C (**a**,**b**), 35 °C (**c**,**d**), 50 °C (**e**,**f**) in TZ-PIM, in pure and mixed-gas conditions (Black squares: pure gas; Red circles: ~10% $CO_2$ mixture; Green triangles: ~30% $CO_2$ mixture; Blue diamonds: ~50% $CO_2$ mixture). Experimental data from [4]. Solid lines represent DMS model predictions obtained using the parameters reported in Table 3 in the main text. Dashed lines are DMS model predictions obtained with parameters reported in Table 1 in the main text.

## 2. Sensitivity Analysis of the Dual Mode Sorption Model Predictions of $CO_2$ and $CH_4$ Sorption in PIM-1 in Multicomponent Conditions

### *2.1. Effect of $b_{CO_2}$ on the Calculated Mixed-Gas Sorption of $CH_4$ in PIM-1*

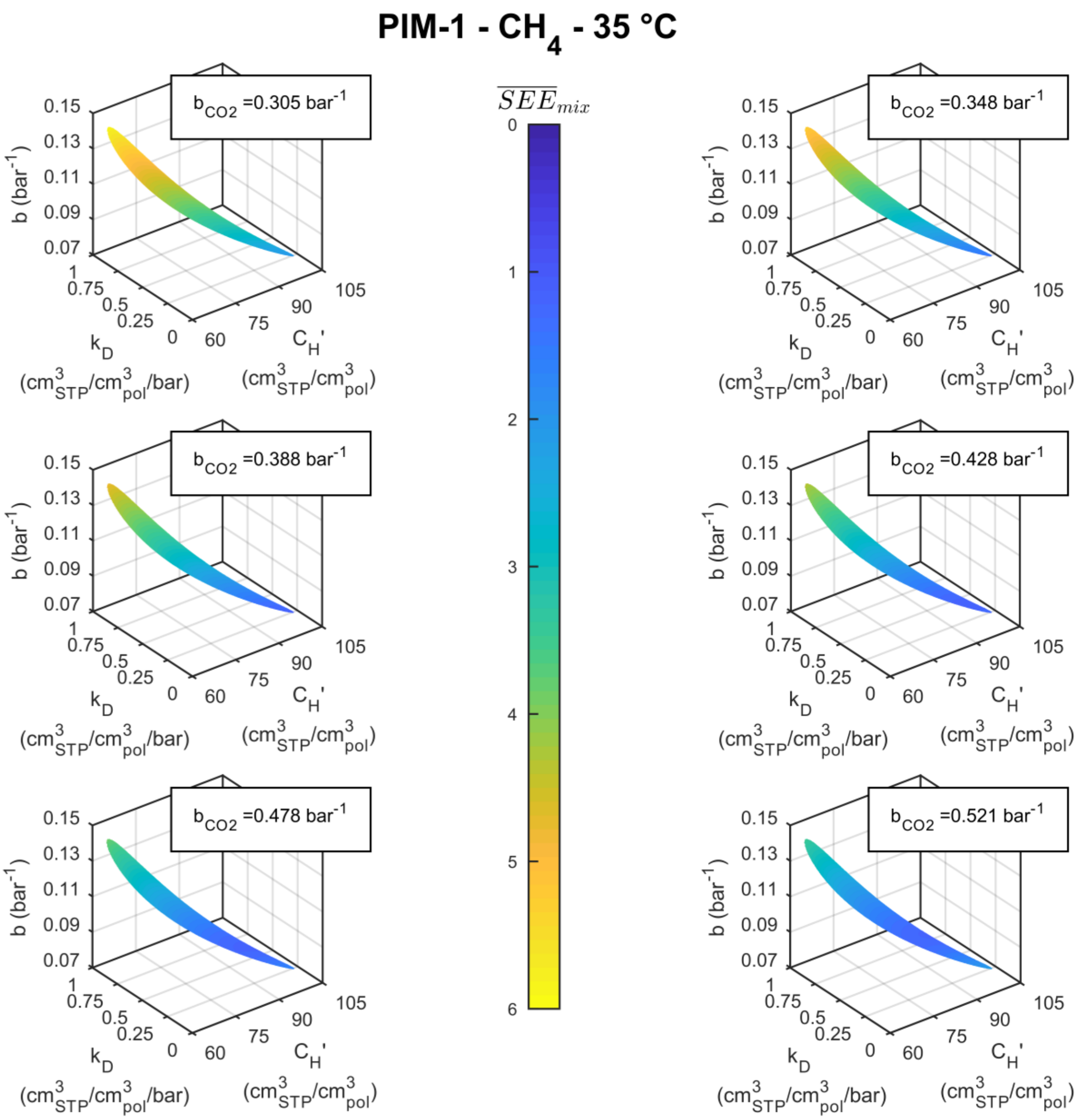


**Figure S6.** Isosurfaces the DMS model parameter space for $CH_4$ sorption in PIM-1 at 35 °C corresponding to $SEE_{pure}$ < $SEE_{max}$, coloured according to the average $SEE_{mix}$ obtained with different values within the confidence interval of $b_{CO_2}$.

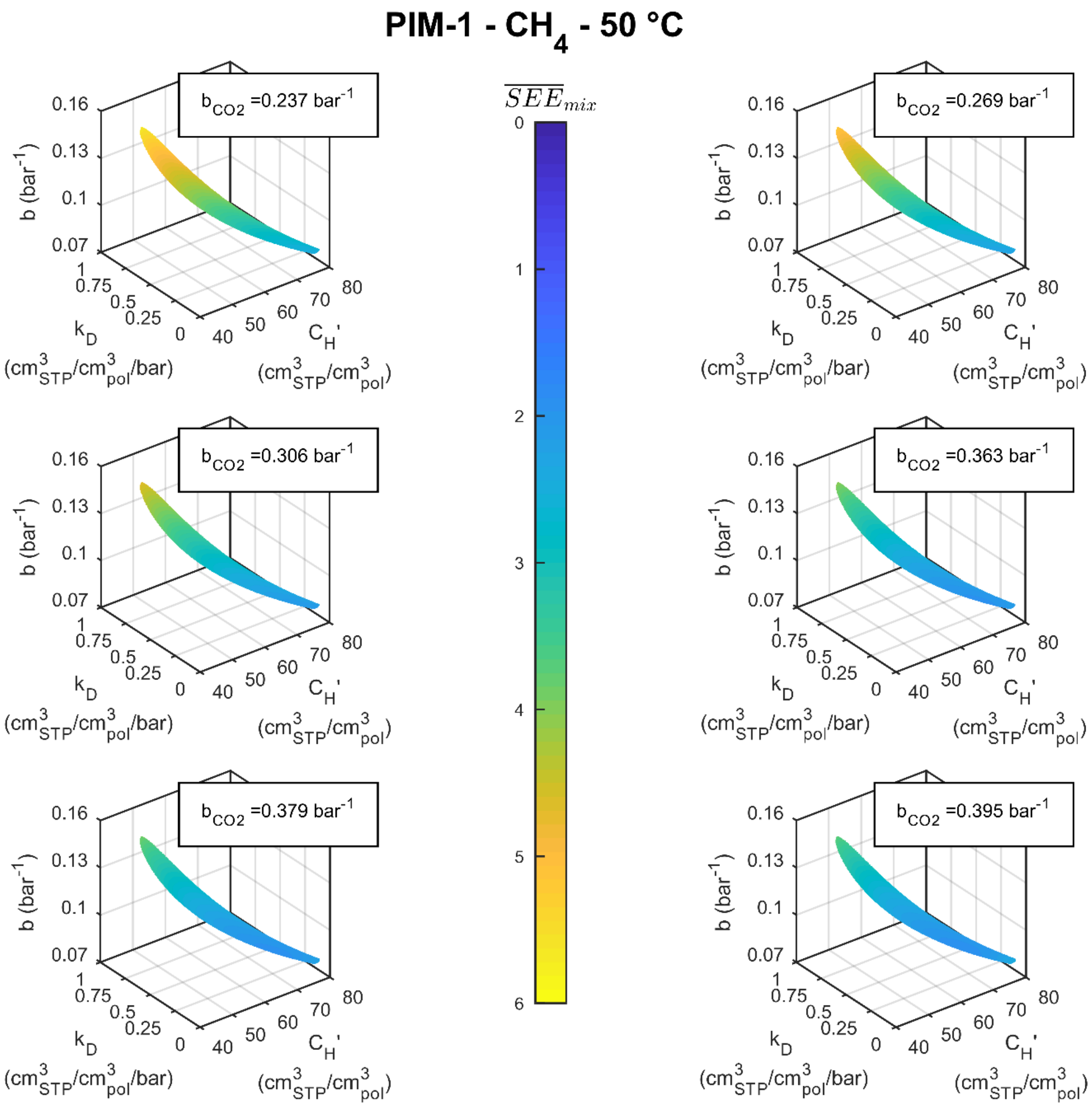


**Figure S7.** Isosurfaces the DMS model parameter space for $CH_4$ sorption in PIM-1 at 50 °C corresponding to $SEE_{pure} < SEE_{max}$, coloured according to the average $SEE_{mix}$ obtained with different values within the confidence interval of $b_{CO_2}$.

*2.2. Confidence Intervals of $CO_2$/PIM-1 DMS Model Parameters*

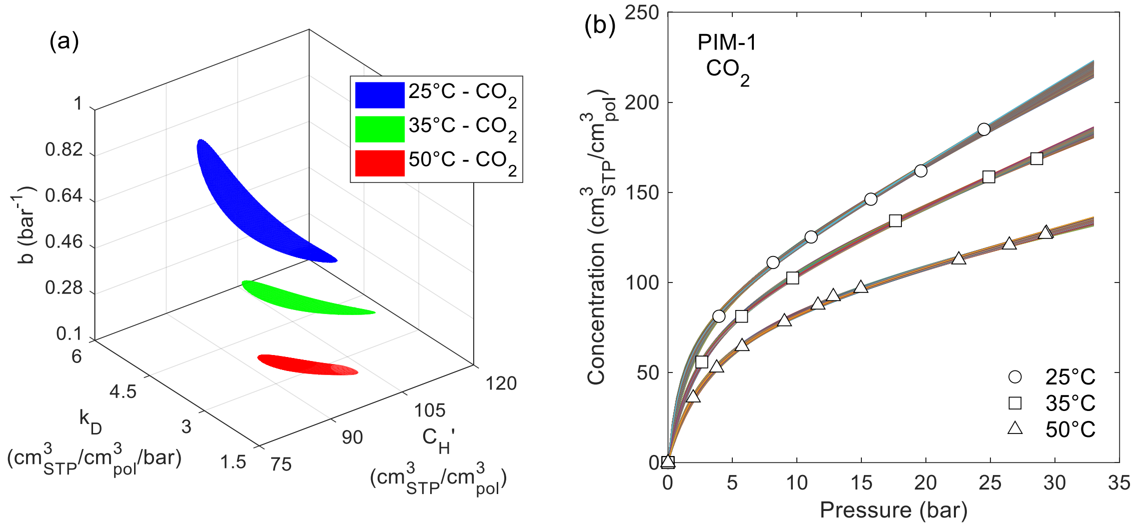


**Figure S8.** (**a**) Surfaces enclosing the range where DMS parameter sets yield *SEE* < *SEE<sub>max</sub>* in the prediction of $CO_2$ sorption in PIM-1 at three different temperatures; (**b**) $CO_2$ sorption isotherms in PIM-1 at 25, 35 and 50 °C, calculated with all the parameter sets enclosed by the corresponding coloured regions in the plot on the left.

**Table S1.** Confidence intervals of the fugacity-based DMS parameters yielding and average relative deviation < 1.5% in the calculation of $CO_2$ sorption in PIM-1 at three different temperatures.

| T (°C) | $k_{D,CO_2}$ $\left(\frac{cm^3_{STP}}{cm^3_{pol}bar}\right)$ | $C'_{H,CO_2}$ $\left(\frac{cm^3_{STP}}{cm^3_{pol}}\right)$ | $b_{CO_2}$ $(bar^{-1})$ |
|---|---|---|---|
| 25 | $4.046^{+0.253}_{-0.552}$ | $90.04^{+16.57}_{-6.50}$ | $0.710^{+0.272}_{-0.291}$ |
| 35 | $2.890^{+0.311}_{-0.308}$ | $94.83^{+12.53}_{-10.15}$ | $0.388^{+0.133}_{-0.083}$ |
| 50 | $1.596^{+0.325}_{-0.096}$ | $89.30^{+6.47}_{-10.88}$ | $0.290^{+0.105}_{-0.053}$ |

*2.3. Uncertainty in Mixed-Gas Sorption of $CO_2$ in PIM-1*

Set 1 and Set 2 reported in Table S2 correspond, respectively, to the highest and lowest accuracy in the prediction of mixed-gas sorption of $CO_2$ in PIM-1, among all the parameter sets belonging to the confidence regions displayed in Figure S8.

**Table S2.** DMS model fugacity-based parameter sets used in the calculation of mixed-gas sorption of $CO_2$ in PIM-1 reported in Figure S9.

| | T (°C) | $k_{D,CO_2}$ $\left(\frac{cm^3_{STP}}{cm^3_{pol}bar}\right)$ | $C'_{H,CO_2}$ $\left(\frac{cm^3_{STP}}{cm^3_{pol}}\right)$ | $b_{CO_2}$ $(bar^{-1})$ | $SEE_{pure}$ $\left(\frac{cm^3_{STP}}{cm^3_{pol}bar}\right)$ | $\overline{SEE}_{mix}$ $\left(\frac{cm^3_{STP}}{cm^3_{pol}bar}\right)$ |
|---|---|---|---|---|---|---|
| | 25 | 4.284 | 84.93 | 0.822 | 1.998 | 2.41 |
| Set 1 | 35 | 3.156 | 85.69 | 0.518 | 1.791 | 2.28 |
| | 50 | 1.906 | 78.01 | 0.395 | 1.498 | 1.92 |
| | 25 | 3.532 | 105.85 | 0.413 | 1.999 | 6.00 |
| Set 2 | 35 | 2.599 | 105.40 | 0.299 | 1.797 | 4.93 |
| | 50 | 1.500 | 94.87 | 0.238 | 1.482 | 2.77 |

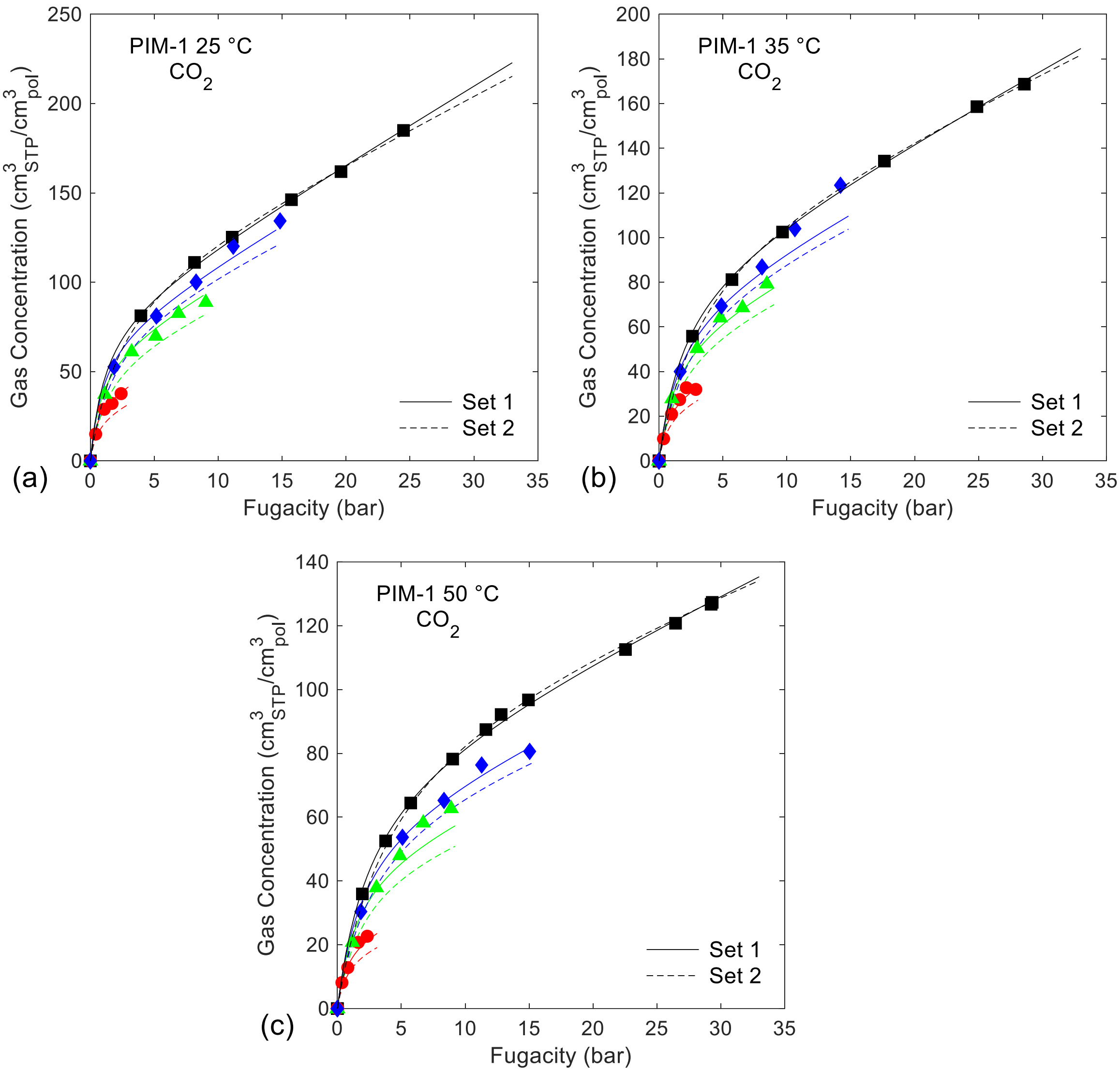


**Figure S9.** Dual Mode Sorption model mixed-gas predictions of $CO_2$ sorption in PIM-1 at 25 °C (**a**), 35 °C (**b**), 50 °C (**c**) obtained with the two parameter sets reported in Table S2.

*2.4. Effect of $b_{CH_4}$ on the Calculated Mixed-Gas Sorption of CO2 in PIM-1*

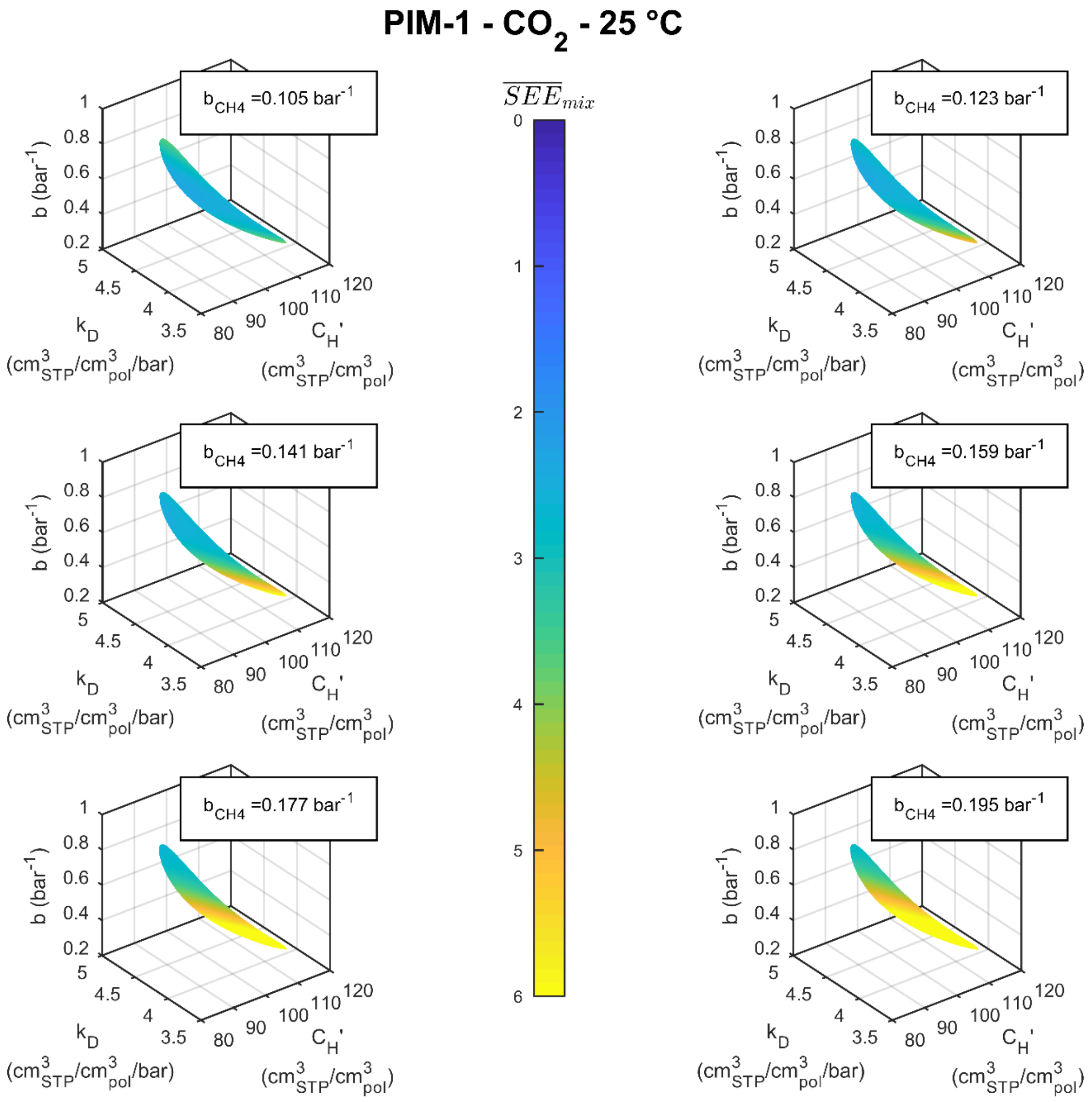


**Figure S10.** Isosurfaces the DMS model parameter space for CO2 sorption in PIM-1 at 25 °C corresponding to $SEE_{pure} < SEE_{max}$, coloured according to the average $SEE_{mix}$ obtained with different values within the confidence interval of $b_{CH_4}$.

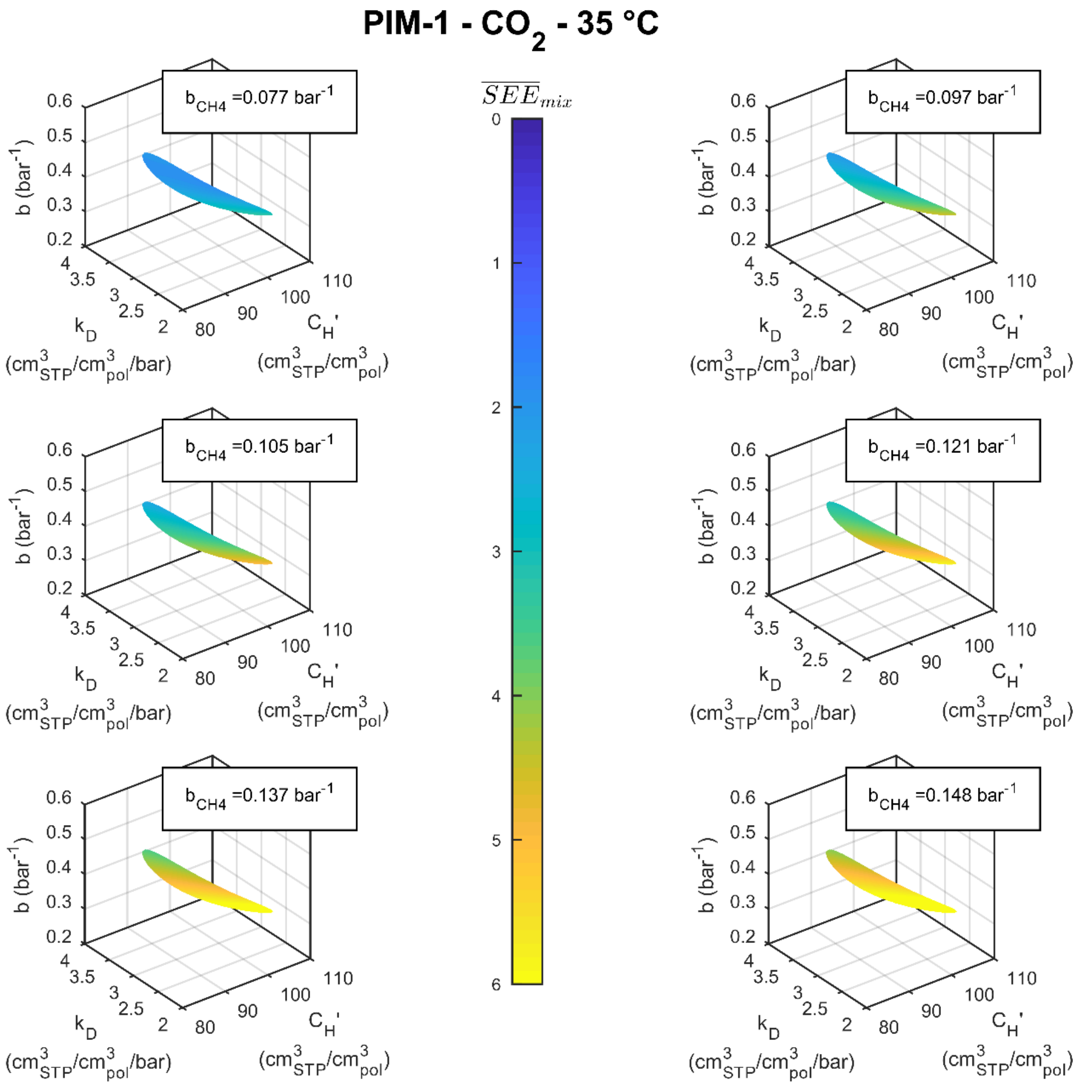


**Figure S11.** Isosurfaces the DMS model parameter space for $CO_2$ sorption in PIM-1 at 35 °C corresponding to $SEE_{pure} < SEE_{max}$, coloured according to the average $SEE_{mix}$ obtained with different values within the confidence interval of $b_{CH_4}$.

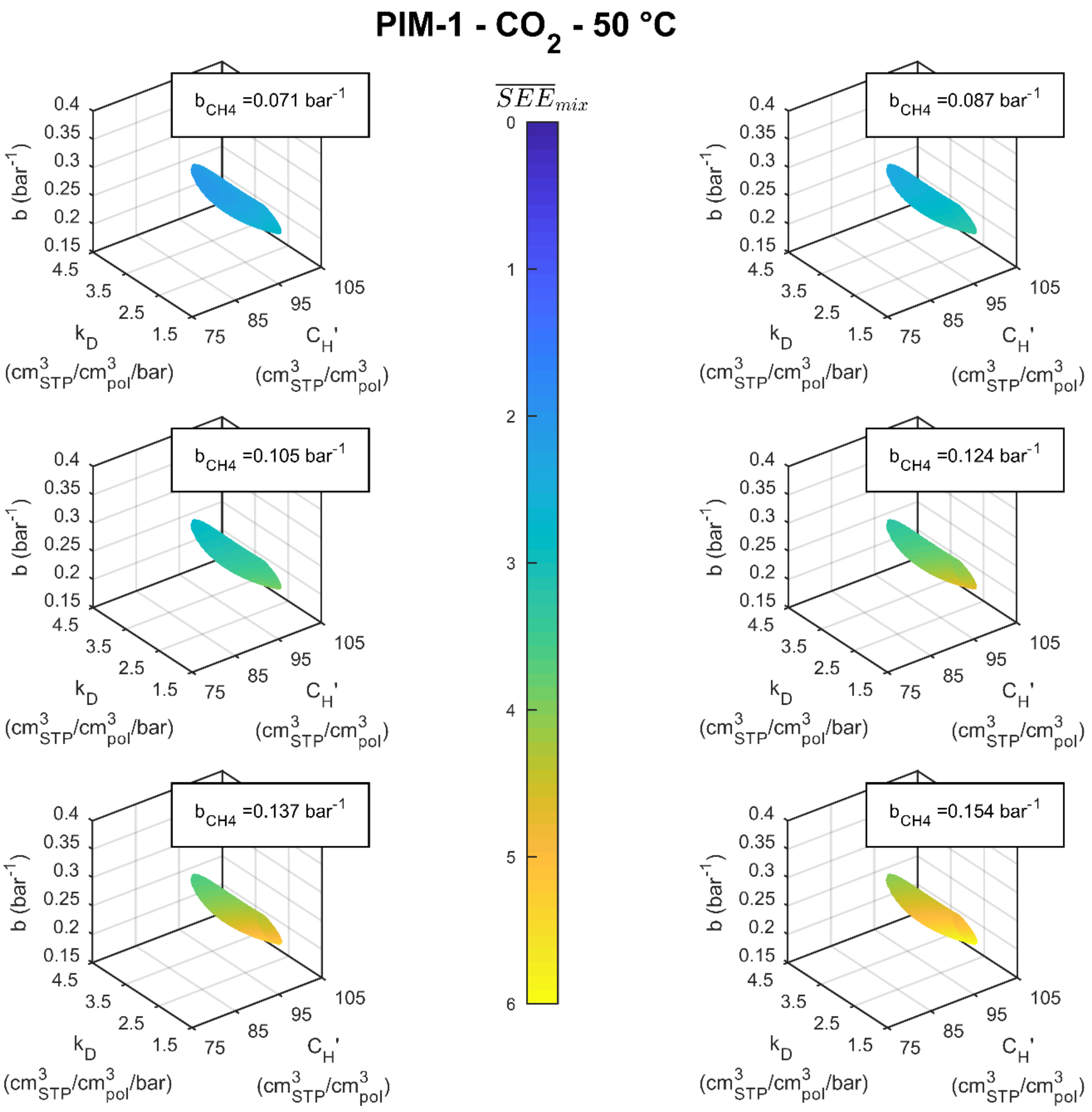


**Figure S12.** Isosurfaces in the DMS model parameter space for $CO_2$ sorption in PIM-1 at 50 °C corresponding to $SEE_{pure} < SEE_{max}$, coloured according to the average $SEE_{mix}$ obtained with different values within the confidence interval of $b_{CH_4}$.

## 3. Sensitivity Analysis of the Dual Mode Sorption Model Predictions of $CO_2$ and $CH_4$ Sorption in TZ-PIM in Multicomponent Conditions

### *3.1. Confidence Intervals of $CO_2$/TZ-PIM and $CH_4$/TZ-PIM DMS Model Parameters*

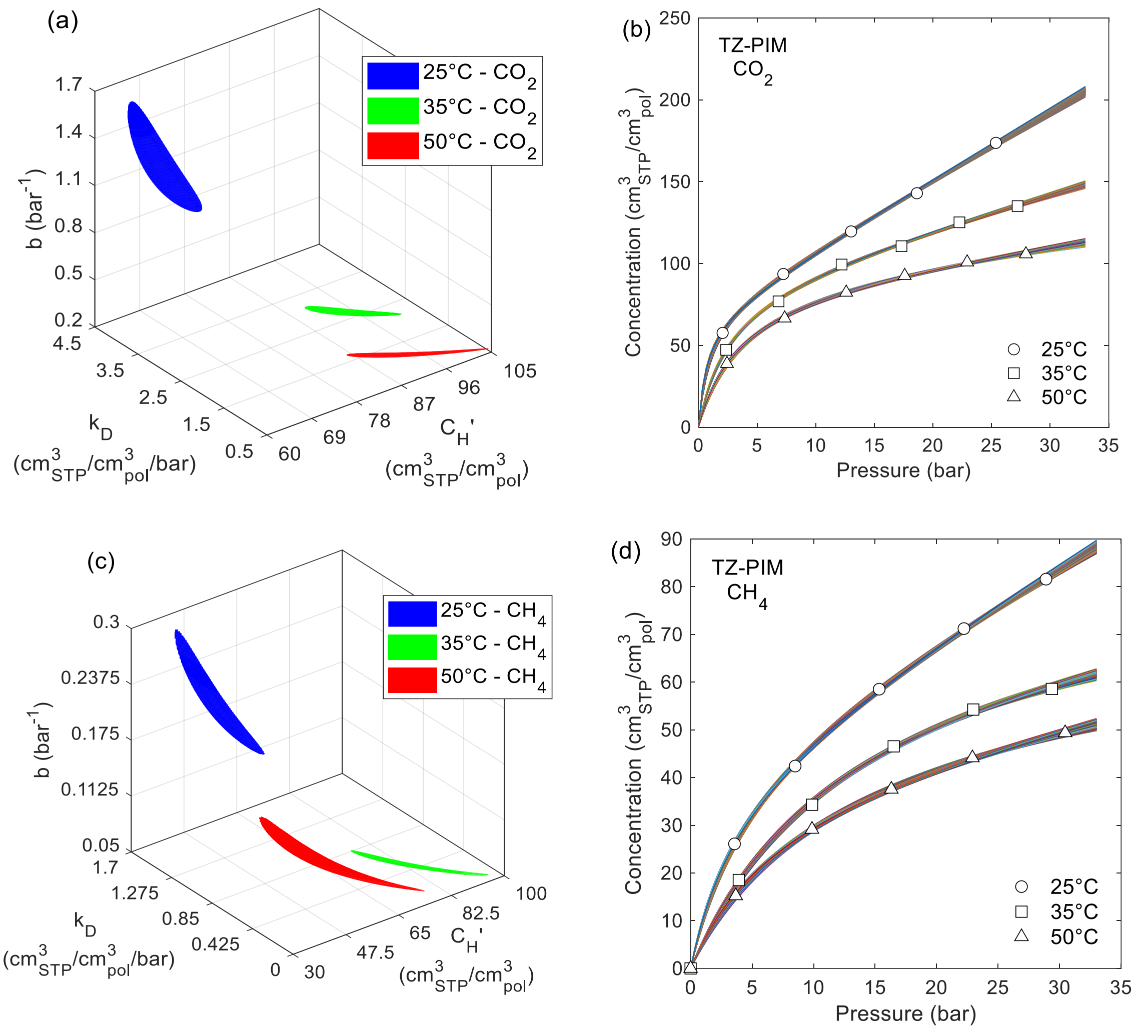


**Figure S13.** Surfaces enclosing the range where DMS parameter sets yield *SEE* < *SEE$_{max}$* in the prediction of (**a**) $CO_2$ and (**c**) $CH_4$ sorption in TZ-PIM at three different temperatures; (**b**) $CO_2$ and (**d**) $CH_4$ sorption isotherms in TZ-PIM calculated with all the parameter sets enclosed by the corresponding coloured region in the plot on the left.

**Table S3.** Confidence intervals of the fugacity-based DMS parameters (average relative deviation < 1.5%) for $CO_2$ and $CH_4$ sorption in TZ-PIM at three different temperatures.

| $T$ (°C) | $k_{D,CO_2}$ $\left(\frac{cm^3_{STP}}{cm^3_{pol}bar}\right)$ | $C'_{H,CO_2}$ $\left(\frac{cm^3_{STP}}{cm^3_{pol}}\right)$ | $b_{CO_2}$ $(bar^{-1})$ | $k_{D,CH_4}$ $\left(\frac{cm^3_{STP}}{cm^3_{pol}bar}\right)$ | $C'_{H,CH_4}$ $\left(\frac{cm^3_{STP}}{cm^3_{pol}}\right)$ | $b_{CH_4}$ $(bar^{-1})$ |
|---|---|---|---|---|---|---|
| 25 | $4.127^{+0.224}_{-0.234}$ | $70.58^{+5.75}_{-5.28}$ | $1.127^{+0.474}_{-0.278}$ | $1.400^{+0.191}_{-0.226}$ | $48.09^{+9.50}_{-7.13}$ | $0.214^{+0.080}_{-0.055}$ |
| 35 | $1.982^{+0.226}_{-0.255}$ | $89.53^{+8.15}_{-7.18}$ | $0.378^{+0.084}_{-0.062}$ | $0.378^{+0.066}_{-0.378}$ | $67.12^{+27.58}_{-3.99}$ | $0.087^{+0.012}_{-0.029}$ |
| 50 | $0.903^{+0.307}_{-0.346}$ | $92.42^{+12.85}_{-10.35}$ | $0.263^{+0.070}_{-0.055}$ | $0.350^{+0.244}_{-0.292}$ | $51.41^{+22.33}_{-13.26}$ | $0.101^{+0.047}_{-0.035}$ |

### 3.2. Uncertainty in Mixed-Gas Sorption of $CO_2$ and $CH_4$ in TZ-PIM

Set 1 and Set 2 reported in Table S3 correspond, respectively, to the highest and lowest accuracy in the prediction of mixed-gas sorption of $CO_2$ and $CH_4$ in TZ-PIM, among all the parameter sets belonging to the confidence regions displayed in Figure S13.

**Table S4.** DMS model fugacity-based parameter sets used in the calculation of mixed-gas sorption of $CO_2$ and $CH_4$ in TZ-PIM reported in Figure S14.

| | **T** (°C) | $k_{D,CO_2}$ $\left(\frac{cm^3_{STP}}{cm^3_{pol}bar}\right)$ | $C'_{H,CO_2}$ $\left(\frac{cm^3_{STP}}{cm^3_{pol}}\right)$ | $b_{CO_2}$ $(bar^{-1})$ | $SEE_{pure}$ $\left(\frac{cm^3_{STP}}{cm^3_{pol}bar}\right)$ | $\overline{SEE}_{mix}$ $\left(\frac{cm^3_{STP}}{cm^3_{pol}bar}\right)$ |
|---|---|---|---|---|---|---|
| | 25 | 4.179 | 70.03 | 1.270 | 1.993 | 7.62 |
| Set 1 | 35 | 2.150 | 84.07 | 0.454 | 1.898 | 5.16 |
| | 50 | 1.153 | 83.35 | 0.333 | 1.591 | 7.60 |
| | 25 | 3.961 | 75.19 | 0.849 | 1.995 | 9.35 |
| Set 2 | 35 | 1.807 | 95.82 | 0.316 | 1.896 | 7.50 |
| | 50 | 0.626 | 103.55 | 0.209 | 1.597 | 11.07 |
| | **T** (°C) | $k_{D,CH_4}$ $\left(\frac{cm^3_{STP}}{cm^3_{pol}bar}\right)$ | $C'_{H,CH_4}$ $\left(\frac{cm^3_{STP}}{cm^3_{pol}}\right)$ | $b_{CH_4}$ $(bar^{-1})$ | $SEE_{pure}$ $\left(\frac{cm^3_{STP}}{cm^3_{pol}bar}\right)$ | $\overline{SEE}_{mix}$ $\left(\frac{cm^3_{STP}}{cm^3_{pol}bar}\right)$ |
| | 25 | 1.393 | 47.23 | 0.239 | 1.049 | 1.83 |
| Set 1 | 35 | 0.280 | 73.01 | 0.081 | 0.947 | 1.14 |
| | 50 | 0.010 | 71.73 | 0.067 | 0.798 | 2.17 |
| | 25 | 1.174 | 57.59 | 0.160 | 1.068 | 3.73 |
| Set 2 | 35 | 0.071 | 93.98 | 0.058 | 0.949 | 3.34 |
| | 50 | 0.594 | 30.715 | 0.152 | 0.799 | 5.63 |

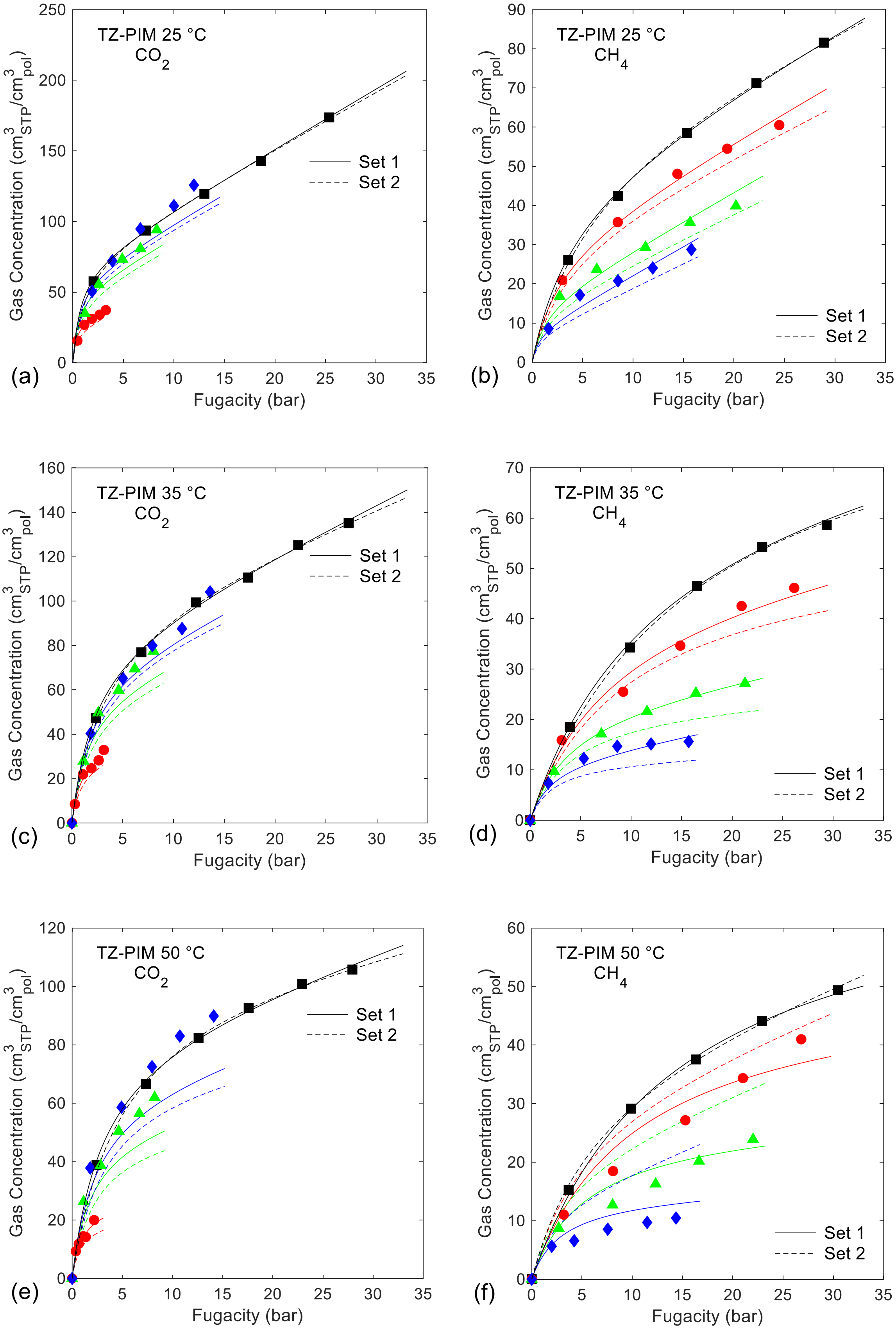


**Figure S14.** Dual Mode Sorption model mixed-gas predictions of $CO_2$ and $CH_4$ at 25 °C (**a,b**), 35 °C (**c,d**), 50 °C (**e,f**) in TZ-PIM, obtained with the two parameter sets reported in in Table S3. Solid lines are obtained with Set 1, dashed ones with Set 2.

### *3.3. Effect of $b_{CO_2}$ on the Calculated Mixed-Gas Sorption of $CH_4$ in TZ-PIM*

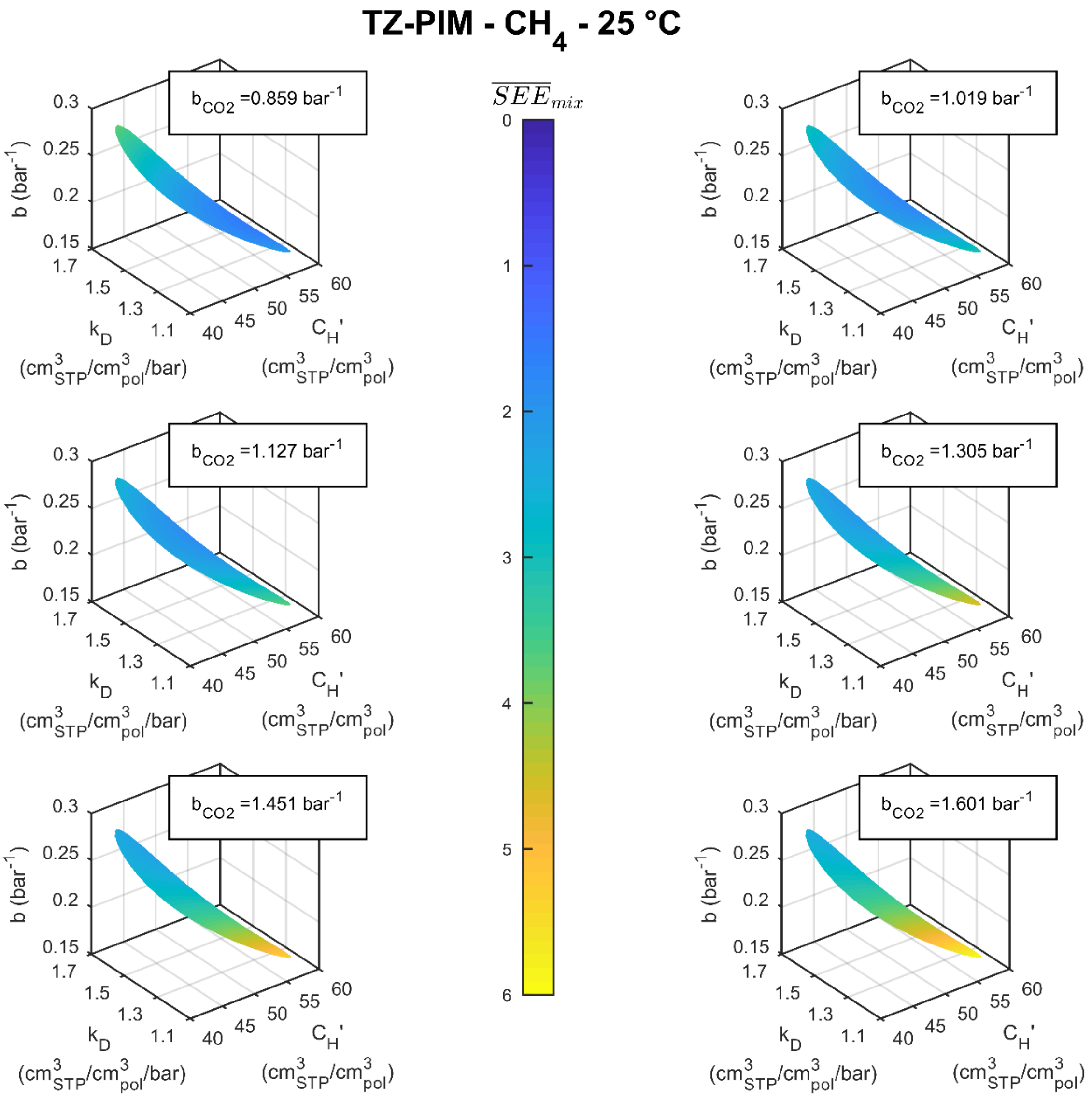


**Figure S15.** Isosurfaces in the DMS model parameter space for $CH_4$ sorption in TZ-PIM at 25 °C corresponding to $SEE_{pure} < SEE_{max}$, coloured according to the average $SEE_{mix}$ obtained with different values within the confidence interval of $b_{CO_2}$.

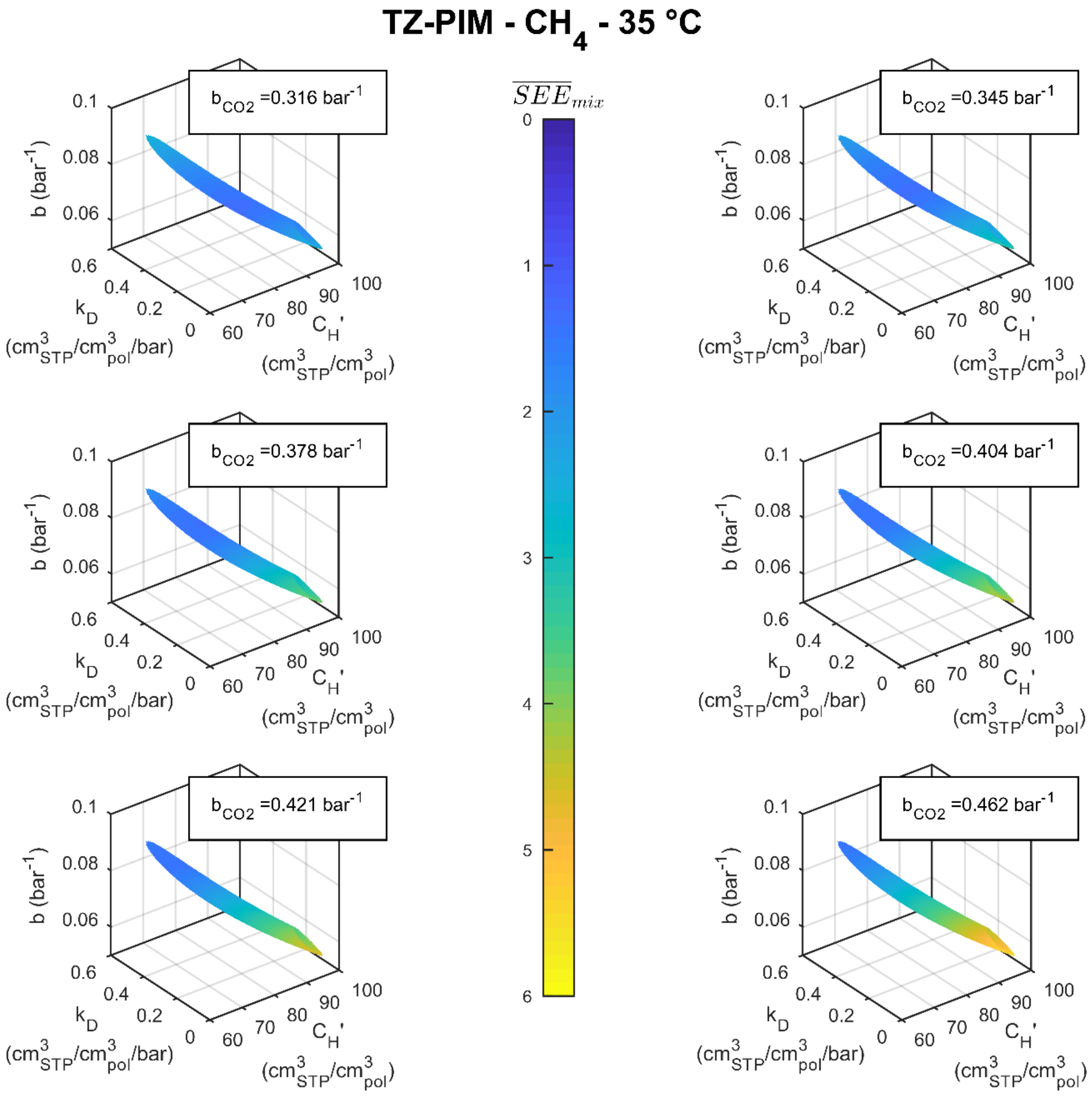


**Figure S16.** Isosurfaces in the DMS model parameter space for $CH_4$ sorption in TZ-PIM at 35 °C corresponding to $SEE_{pure} < SEE_{max}$, coloured according to the average $SEE_{mix}$ obtained with different values within the confidence interval of $b_{CO_2}$.

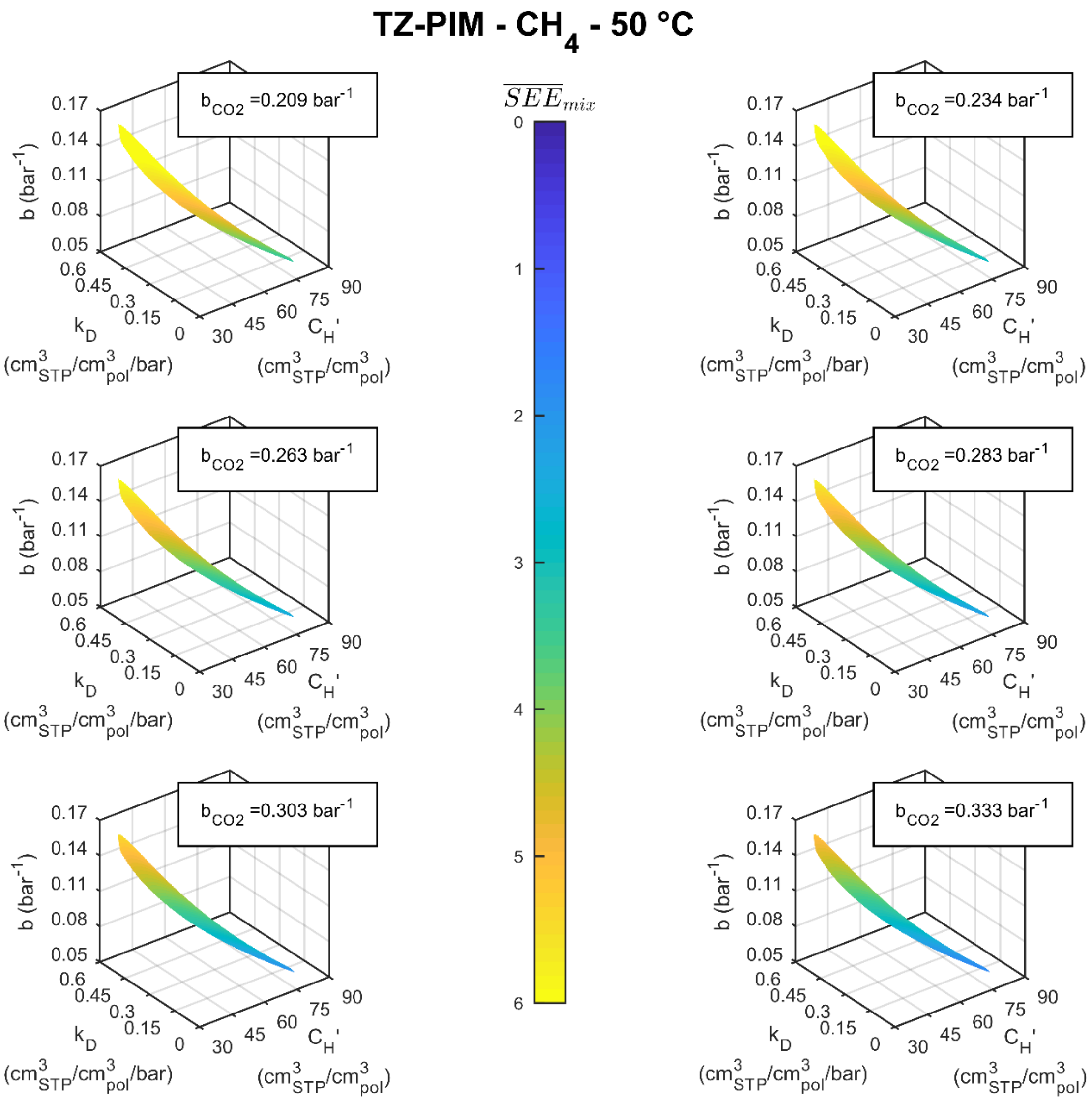


**Figure S17.** Isosurfaces in the DMS model parameter space for $CH_4$ sorption in TZ-PIM at 50 °C corresponding to $SEE_{pure} < SEE_{max}$, coloured according to the average $SEE_{mix}$ obtained with different values within the confidence interval of $b_{CO_2}$.

### *3.4. Effect of $b_{CH_4}$ on the Calculated Mixed-Gas Sorption of $CO_2$ in PIM-1*

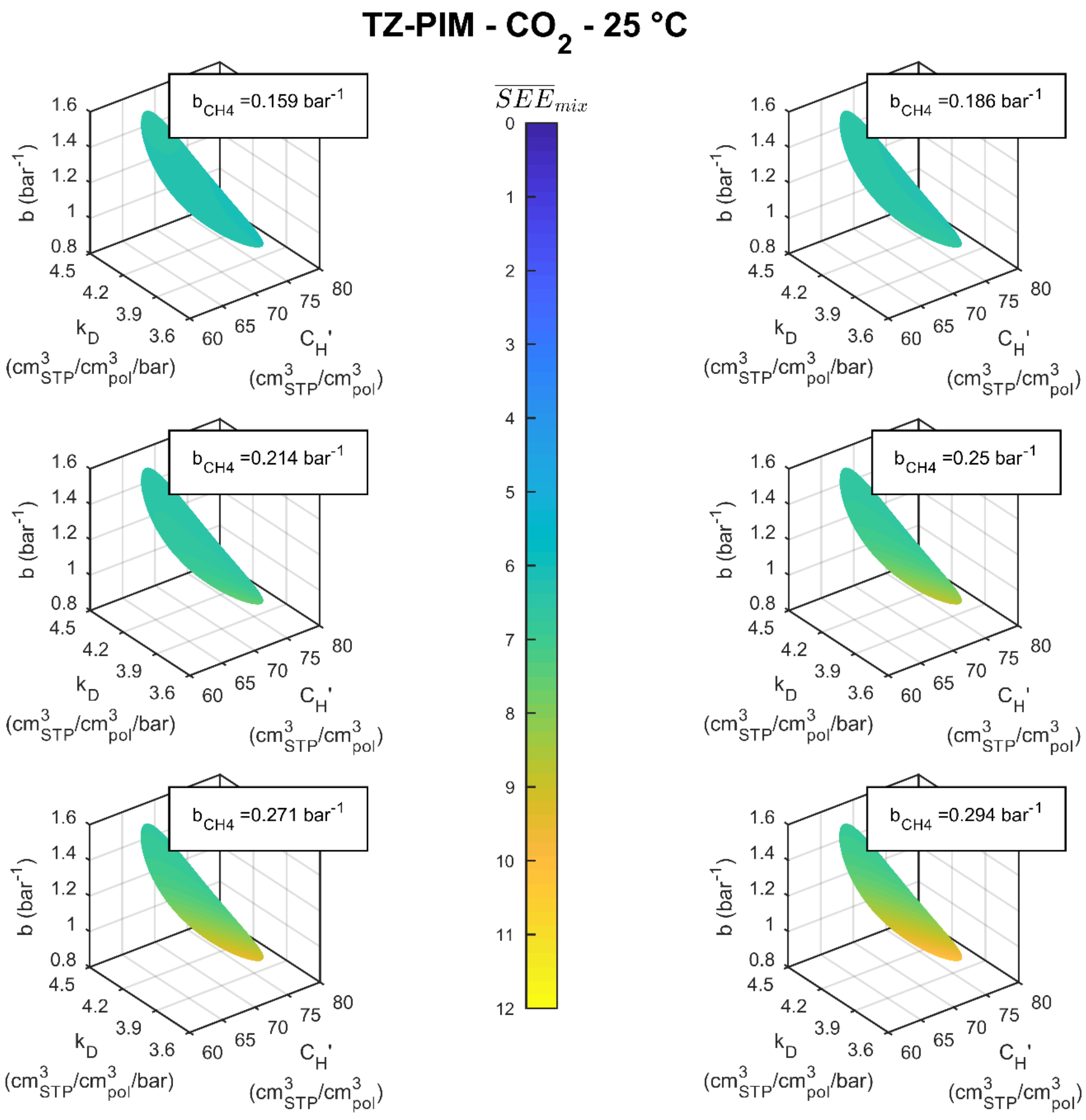


**Figure S18.** Isosurfaces in the DMS model parameter space for $CO_2$ sorption in TZ-PIM at 25 °C corresponding to $SEE_{pure} < SEE_{max}$, coloured according to the average $SEE_{mix}$ obtained with different values within the confidence interval of $b_{CH_4}$.

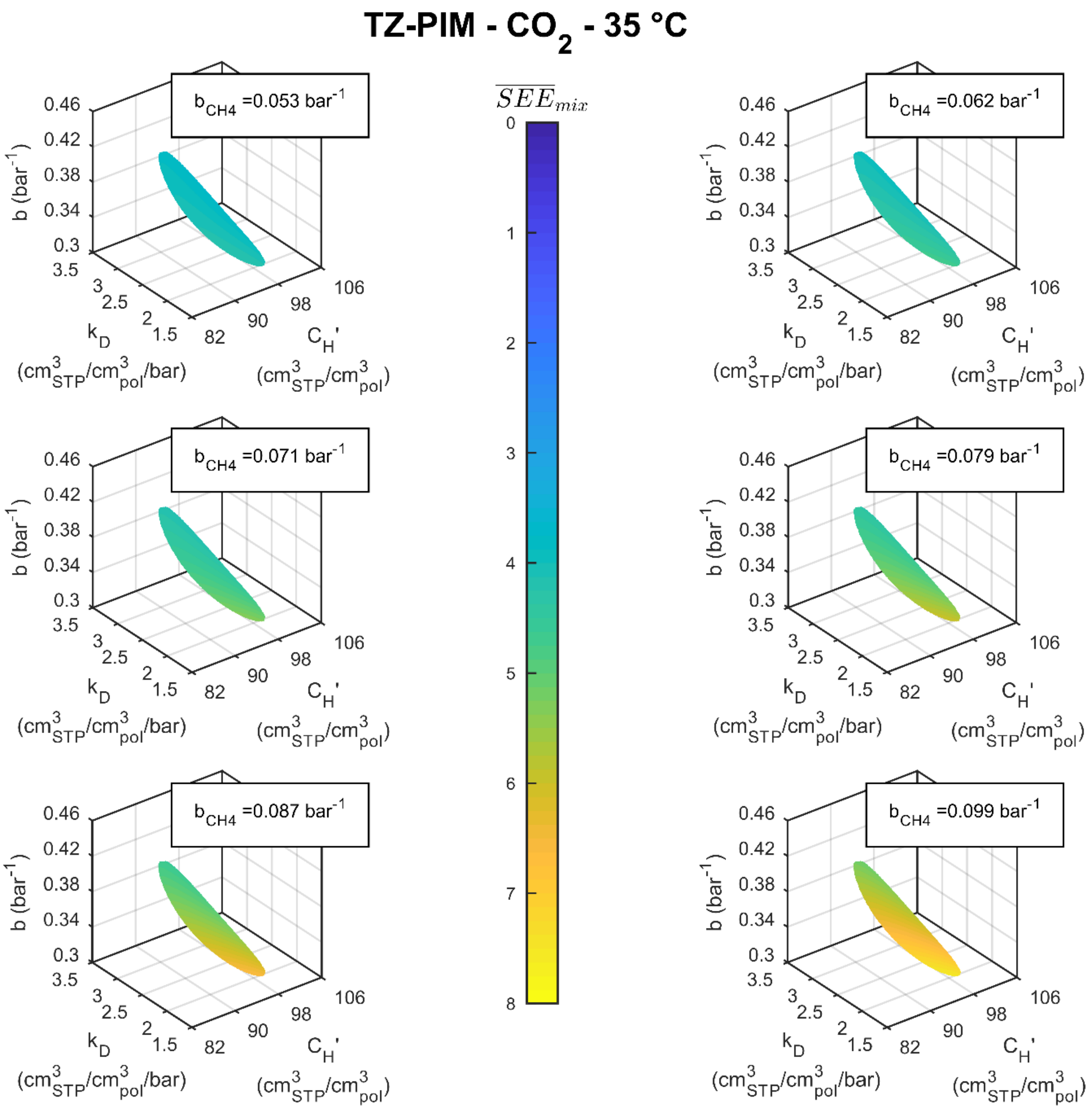


**Figure S19.** Isosurfaces in the DMS model parameter space for $CO_2$ sorption in TZ-PIM at 35 °C corresponding to $SEE_{pure} < SEE_{max}$, coloured according to the average $SEE_{mix}$ obtained with different values within the confidence interval of $b_{CH_4}$.

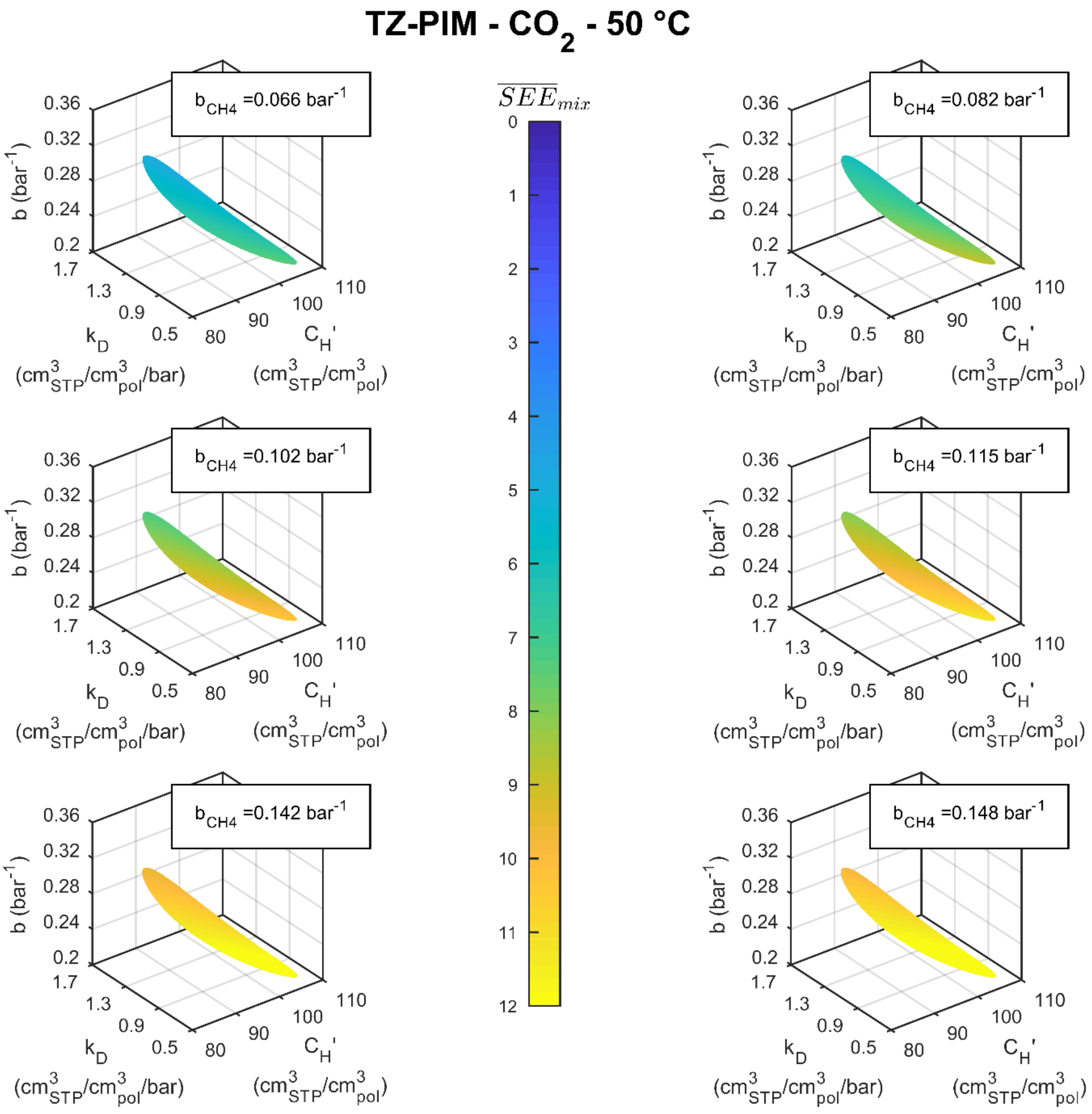


**Figure S20.** Isosurfaces in the DMS model parameter space for $CO_2$ sorption in TZ-PIM at 50 °C corresponding to $SEE_{pure} < SEE_{max}$, coloured according to the average $SEE_{mix}$ obtained with different values within the confidence interval of $b_{CH_4}$.

## References


1. Vopička, O.; De Angelis, M.G.; Sarti, G.C. Mixed gas sorption in glassy polymeric membranes: I. $CO_2/CH_4$ and n-$C_4/CH_4$ mixtures sorption in poly(1-trimethylsilyl-1-propyne) (PTMSP). *J. Membr. Sci.* **2013**, *449*, 97–108, doi:10.1016/j.memsci.2013.06.065.
2. Vopička, O.; De Angelis, M.G.; Du, N.; Li, N.; Guiver, M.D.; Sarti, G.C. Mixed gas sorption in glassy polymeric membranes: II. $CO_2/CH_4$ mixtures in a polymer of intrinsic microporosity (PIM-1). *J. Membr. Sci.* **2014**, *459*, 264–276, doi:10.1016/j.memsci.2014.02.003.
3. Gemeda, A.E.; De Angelis, M.G.; Du, N.; Li, N.; Guiver, M.D.; Sarti, G.C. Mixed gas sorption in glassy polymeric membranes. III. $CO_2/CH_4$ mixtures in a polymer of intrinsic microporosity (PIM-1): Effect of temperature. *J. Membr. Sci.* **2017**, *524*, 746–757, doi:10.1016/j.memsci.2016.11.053.
4. Ricci, E.; Gemeda, A.E.; Du, N.; Li, N.; De Angelis, M.G.; Guiver, M.D.; Sarti, G.C. Sorption of the $CO_2/CH_4$ mixture in TZ-PIM, PIM-1 and PTMSP: experimental data and NELF-model based analysis of competitive sorption and its impact on the selectivity. *J. Membr. Sci.* **2018**, submitted for publication.

© 2018 by the authors. Submitted for possible open access publication under the terms and conditions of the Creative Commons Attribution (CC BY) license (http://creativecommons.org/licenses/by/4.0/).
